# Dietary intake of micronized avian eggshell membrane in aged mice reduces circulating inflammatory markers, increases microbiota diversity, and attenuates skeletal muscle aging

**DOI:** 10.3389/fnut.2023.1336477

**Published:** 2024-01-15

**Authors:** Sissel Beate Rønning, Harald Carlsen, Sérgio Domingos Cardoso Rocha, Ida Rud, Nina Solberg, Vibeke Høst, Eva Veiseth-Kent, Henriette Arnesen, Silje Bergum, Bente Kirkhus, Ulrike Böcker, Nada Abedali, Amanda Rundblad, Pia Bålsrud, Ingrid Måge, Kirsten Bjørklund Holven, Stine Marie Ulven, Mona Elisabeth Pedersen

**Affiliations:** ^1^Nofima AS, Food Division, Ås, Norway; ^2^Faculty of Chemistry, Biotechnology and Food Science, Norwegian University of Life Sciences, Ås, Norway; ^3^Faculty of Biosciences, Norwegian University of Life Sciences, Ås, Norway; ^4^Faculty of Veterinary Medicine, Norwegian University of Life Sciences, Ås, Norway; ^5^Department of Nutrition, Institute of Basic Medical Sciences, University of Oslo, Oslo, Norway; ^6^National Advisory Unit on Familial Hypercholesterolemia, Department of Endocrinology, Morbid Obesity and Preventive Medicine, Oslo University Hospital, Oslo, Norway

**Keywords:** eggshell membrane, protein digestibility, microbiota, inflammatory responses, skeletal muscle aging

## Abstract

**Introduction:**

Avian eggshell membrane (ESM) is a complex extracellular matrix comprising collagens, glycoproteins, proteoglycans, and hyaluronic acid. We have previously demonstrated that ESM possesses anti-inflammatory properties *in vitro* and regulates wound healing processes *in vivo*. The present study aimed to investigate if oral intake of micronized ESM could attenuate skeletal muscle aging associated with beneficial alterations in gut microbiota profile and reduced inflammation.

**Methods:**

Elderly male C57BL/6 mice were fed an AIN93G diet supplemented with 0, 0.1, 1, or 8% ESM. Young mice were used as reference. The digestibility of ESM was investigated using the static *in vitro* digestion model INFOGEST for older people and adults, and the gut microbiota profile was analyzed in mice. In addition, we performed a small-scale pre-clinical human study with healthy home-dwelling elderly (>70 years) who received capsules with a placebo or 500 mg ESM every day for 4 weeks and studied the effect on circulating inflammatory markers.

**Results and discussion:**

Intake of ESM in elderly mice impacted and attenuated several well-known hallmarks of aging, such as a reduction in the number of skeletal muscle fibers, the appearance of centronucleated fibers, a decrease in type IIa/IIx fiber type proportion, reduced gene expression of satellite cell markers *Sdc3* and *Pax7* and increased gene expression of the muscle atrophy marker *Fbxo32.* Similarly, a transition toward the phenotypic characteristics of young mice was observed for several proteins involved in cellular processes and metabolism. The digestibility of ESM was poor, especially for the elderly condition. Furthermore, our experiments showed that mice fed with 8% ESM had increased gut microbiota diversity and altered microbiota composition compared with the other groups. ESM in the diet also lowered the expression of the inflammation marker TNFA in mice and *in vitro* in THP-1 macrophages. In the human study, intake of ESM capsules significantly reduced the inflammatory marker CRP. Altogether, our results suggest that ESM, a natural extracellular biomaterial, may be attractive as a nutraceutical candidate with a possible effect on skeletal muscle aging possibly through its immunomodulating effect or gut microbiota.

## 1 Introduction

Biological changes, like loss or change in type of muscle fibers (muscle atrophy), resulting in reduced muscle strength, are often observed in skeletal muscle during aging (1, 2). Skeletal muscle mass has been reported to be constant until age 40, followed by a slow but continuous loss of muscle that becomes more pronounced by age 70 (2). Skeletal muscle mass is maintained by balancing protein synthesis and degradation. A shift in this balance, with more significant protein degradation than synthesis, can result in muscle atrophy. Old muscles display a severely perturbed protein expression pattern involving enzymes of various metabolic pathways, mitochondrial abnormalities, ion homeostasis regulatory components, and cellular stress response elements (3). In addition, the regenerating capacity of skeletal muscle stem cells (MuSCs) is diminished, and fewer MuSCs are activated upon injury and exercise to build new muscle fibers. Morphologically, an increased number of centrally nucleated fibers is detected in the skeletal muscle during aging (1, 4), reflecting earlier cycles of degeneration and regeneration. In many conditions, muscle atrophy is associated with chronic elevations in circulating inflammatory cytokines, particularly TNFA, which disturb energy balance (5). During skeletal muscle regeneration, MuSCs are typically resident within the muscular structure. Upon activation, they re-enter the cell cycle and proliferate as myoblasts, eventually undergoing terminal differentiation into myocytes that fuse into pre-existing damaged muscle fibers or fuse, generating new muscle fibers. During regeneration, a portion of MuSCs self-renew to maintain a pool of quiescent MuSCs that express Paired box protein 7 (PAX7) but no other myogenic transcription factors (6). In the elderly, skeletal muscle regeneration is impaired and associated with functional defects of the MuSCs (7). The transmembrane proteoglycans, Syndecan-3 (SDC3) and Syndecan-4 (SDC4), play prominent but distinct roles in this regulation (8–10). SDC3 is especially interesting as its presence is necessary to maintain MuSCs quiescence (8).

There is increasing pre-clinical data and evidence that dietary nutrient supplementation can protect skeletal muscle from atrophy damage (11). Eggshell membrane (ESM) is an interesting biological material in this perspective due to its high content of bioactive compounds. Clinical trials and *in vitro* experiments have shown that ESM has promising benefits for joint health, wound healing, and skin health [reviewed by (12, 13)]. It is a low-cost by-product readily available as a remnant of the chicken egg processing (breaking) industry. ESM is a highly crosslinked-fiber-like structure that contains proteins (14–16) and complex carbohydrates such as glycosaminoglycans (GAGs) (17) and N-glycans (18). ESM has low solubility in water, and hydrolysates of ESM have been investigated for bioactive properties (19–22). Ruff and co-workers demonstrated that the anti-inflammatory properties of ESM hydrolysate are retained after *in vitro* digestion (23). Also, clinical trials indicate that oral intake of ESM hydrolysate can reduce joint pain and stiffness (24). A previous study has shown that 8% micronized ESM (ESM powder) ameliorates intestinal inflammation by facilitating restitution of epithelial injury and alleviates microbial dysbiosis during bowel disease in young mice (25). In that study, intake of ESM powder increased skeletal muscle mass, pointing toward an effect on skeletal muscle homeostasis. However, no further characterization at the molecular level of skeletal muscle was performed in their study. A recent study has shown that ESM prevents murine pre-cachexia in young mice (13). To the best of our knowledge, no studies have investigated the potential of ESM on skeletal muscle ageing. We, therefore, set out to investigate micronized ESM as a potential nutraceutical candidate with an effect on skeletal muscle aging. The present study aimed to investigate if oral intake of ESM could attenuate skeletal muscle aging by affecting gut microbiota and inflammation.

## 2 Materials and methods

### 2.1 Preparation of micronized ESM powder, ESM-carbohydrate, and ESM-peptide fraction

The ESM used in the study was industrial eggshell membrane (NOR-ESM) provided from Norilia, Norway, produced at Nortura Revetal plant using a patented process owned by Biovotec (international application: WO 2015/058790 A1). The NOR-ESM was further washed in 0.1 M HCL, purified in dH_2_0, dried, and milled to small particles under 250 μm. This fraction, called ESM powder in our study, was used both for the *in vitro* cell experiments, mice feeding trial, and the human pilot study. In addition, further preparations of ESM powder were performed for bioactivity studies of ESM constituents in cell experiments as follows: total protein was extracted from ESM powder by magnetic stirring 8 g ESM powder in 100 mL Tris–HCl buffer containing 4% SDS and 5.6 M urea for 4 days at 4°C. The mixture was centrifuged at 10.000 x g for 30 min, then the supernatant was transferred to a new tube and further centrifuged for 30 min at 4.000 x g. The clear supernatant was dialyzed against dH_2_O using dialysis tubes (MwCO 12–14.000 Da; Sigma-Aldrich, United States) for 3 days and then freeze-dried. To remove the protein cores, the lyophilized powder was incubated with 1 mg/mL papain (Sigma-Aldrich) in 0.05 M Tris–HCl pH 7.4 and 5 mM L-cysteine (Sigma-Aldrich) at 37°C for 48 h, with 2 x refreshing with papain during this period. Papain degraded material was dialyzed (MwCO 6.000–8.000 Da; Sigma-Aldrich) against dH_2_O and freeze-fried. This fraction is called carbohydrate fraction and consists of various carbohydrate types, including glycans and GAGs above 6.000 Da. As previously described, the ESM-peptide fraction was prepared using ESM powder subjected to enzymatic digestion by alcalase (26).

### 2.2 Mouse experiment and diets

Fourteen months old (*n* = 60) and three months old (*n* = 15) C57BL/6JRj male mice were purchased from JANVIER LABS (Le Genest-Saint-Isle, France) and housed in individually ventilated cages (Innorack, Innovive, San Diego, CA; *n* = 3 per cage) under controlled conditions (12 h light–dark cycle; 24–26°C, 45–55% relative humidity). Cages were equipped with running wheels, chewing sticks, and igloos, and the mice had free access to water and food. Upon arrival, mice were randomized into their respective groups, acclimatized for 2 weeks, and fed a standard rodent chow diet (RM1, SDS Diet, Essex, United Kingdom) before switching to the experimental diets containing either AIN93G (Control) or ESM powder (described in the section below) mixed into AIN93G (27). To adjust for protein content, casein levels in the ESM diets were reduced by the amount of ESM added to the diets, Table 1. Experimental groups are outlined in Table 2. In brief, five groups were included, four groups with aging mice (14 mo) fed varying ESM content (0, 0.1, 1%, or 8% [where 8% previously shown to have an effect on skeletal muscle mass in young mice with bowl disease] (25)) and one control group with young mice (3 mo) fed only AIN93G control diet. Diets were made by mixing ingredients purchased from Altromin (Altromin, Germany) and pelleted by extrusion (Fôrtek-NMBU, Ås, Norway). The animal experiment was conducted over 10 weeks, and body weight and food intake were recorded weekly. Muscle strength (Grip strength), balance and motor coordination (Rotarod test) were conducted at three time points (Wks 1, 4, and 9). At termination, blood (0.5 mL) was collected by cardiac puncture of mice anesthetized with a cocktail of Zoletil Forte (Virbac, Carros, France), Rompun (Bayer, Oslo, Norway), and Fentadon (Eurovet Animal Health, Bladel, The Netherlands; ZRF; i.p. 0.1 mL ZRF/10 g body weight), with the following active ingredients: Zolezepam (32 mg/kg), Tiletamine (32 mg/kg), Xylazine (4.5 mg/kg), and Fentanyl (26 mg/kg). Mice were immediately euthanized by cervical dislocation after blood collection. To obtain serum, blood was left to clot for 30–45 min in RT and centrifuged (6.000 x g, 10 min, four °C). Serum was stored at −80°C. Left and right tibialis anterior (TA) and gastrocnemius (GTN) were dissected, weighed, and snap-frozen in liquid nitrogen and held at −80°C. Cecum contents for microbiota analysis were snap-frozen and stored at −80°C. The experiment was performed according to European guidelines for the care and use of laboratory animals (European Directive 2010/63/EU) and the Norwegian national guidelines for animal welfare. The protocol was approved by the Norwegian Food Safety Authority (FOTS ID 21576), and the experiment was conducted in the animal facility at the Faculty of Chemistry, Biotechnology and Food Science, Norwegian University of Life Sciences (NMBU; Ås, Norway).

**Table 1:** Mouse diets.

		Eggshell membrane powder		
Ingredients (g/kg)	CONTROL (AIN93G)	0.1%	1%	8%
Casein*	200	199,5	195	164
Cystine, L	3	3	3	3
Starch, Corn	397	397	397	397
Maltodextrin	133	133	133	133
Sucrose	100	100	100	100
Cellulose	50	50	50	50
Soybean Oil, USP	70	70	70	70
MinMix93	35	35	35	35
VitMix93	10	10	10	10
Choline Bitartrate	2,5	2,5	2,5	2,5
ESM*	0	1	10	80

Energy from carbohydrates (65%), proteins (19%) and fat (16%). *Casein was adjusted in the ESM-supplemented diets according to the estimated digestible protein content in the eggshell membrane, which constitutes 46% digestible protein (25). AIN93G, American Institute of Nutrition, 1993, Growth feed. MinMix93, Mineral mixture based on AIN93G. VitMix93, Vitamin mixture based on AIN93G.

**Table 2:** Experimental mice groups.

	Groups-male mice	Age (mo)	Dose ESM	Duration of feeding	n
1	Young	3	0	Ten weeks	15
2	Old	14	0	Ten weeks	15
3	ESM0.1	14	0.1%	Ten weeks	15
4	ESM1	14	1%	Ten weeks	15
5	ESM8	14	8%	Ten weeks	15

### 2.3 Grip-strength and rotarod

We used a Grip strength meter (BIO-GS3, Bioseb, Vitrolles, France) and a Rotarod (Bioseb) to record grip strength and motor coordination. Grip strength was conducted by placing individual mice with all four limbs attached to a grid connected to a Newton meter. The mouse tail was pulled backward until the grip loosened from the grid. The maximal force in g grams was recorded. The test was performed three times with a 1-min interval between each recording and repeated 30 min later for each time point. The Rotarod (Bioseb) measures balance and motor coordination and consists of a rotating beam in which mice are placed, and latency to fall is recorded when mice land on a platform. A protocol allowing a steady increment in rotation speed was used (4–40 rpm in 300 s). For each time point (Weeks 1, 4, and 9), six repetitions of the Rotarod test were carried out over 2 days (3 each day – 45 min recovery between each test).

### 2.4 Real-time qPCR

Tibialis anterior (TA), and gastrocnemius (GA) were collected from old mice (14 mo) fed a regular mouse diet supplemented with 0, 0.1, and 8% ESM for 10 weeks and compared with young mice (3 mo) provided without ESM. The muscles collected were snap-frozen in liquid nitrogen before RNA extraction. RNA was extracted by homogenization in lysis RLT-buffer of RNeasy mini kit (#74104, Qiagen, Hilden, Germany) using a Precellys24 (#74106, Bertin Technologies, Villeurbanne, France) at 5500 rpm for 2×20 s. RNA was further purified following the manufacturer’s protocol of the RNeasy mini kit, including a DNase treatment on column according to the manufacturer’s protocol. RNA from cell cultures (skeletal muscle cells and Caco-2) were also isolated according to the manufacturer’s protocol using the same RNeasy mini kit, including a DNase treatment on column according to the manufacturer’s protocol. According to the manufacturer’s protocol, cDNA was generated from both tissue and cells using TaqMan^®^ Reverse Transcription Reagents (Invitrogen, Carlsbad, CA, United States). RT-qPCR was carried out using a TaqMan Gene expression Master Mix (Life Technologies) and QuantStudio5 (Applied Biosystems, Foster City, CA, United States) PCR System and TaqMan^®^ primer/probe assays (see Table 3 for primer/probes used). Amplification of cDNA by 40 two-step cycles (15 s at 95°C for denaturation of DNA, 1 min at 60°C for primer annealing and extension) was used, and cycle threshold (Ct) values were obtained graphically (Applied Biosystem, Sequence Detection System, Software version 2.2). DCt and DDCt values were calculated according to the MIQE guidelines (28). A comparison of the relative gene expression (RQ) was derived by using the comparative Ct method. In short, values were generated by subtracting ΔCt values between two samples, giving a ΔΔCt value. The relative gene expression (RQ) was then calculated by the formula 2^-ΔΔCt^ (29). Statistical analyses were performed using the ΔΔCt-values.

**Table 3:** Applied biosystem’s primer/probe assays were used in this study.

Gene Target	Species	TaqMan^®^primer/probe assays
*EEF1A1*	*Bos taurus*	Bt03223794_gl
*PAX7*		Hs00242962_ml
*MYOD1*		Bt03244740_ml
*TNFA*		Bt03259156_m1
*NOS2*		Bt03249586_m1
*Sdc4*	*Mus musculus*	Mm00488527_m1
*Sdc3*		Mm01179833_m1
*Col11a2*		Mm00483888_m1
*Col3a1*		Mm00802331_m1
*Pax7*		Mm01354484_m1
*Myod1*		Mm01203489_g1
*Myog*		Mm00446195_g1
*Mstn*		Mm01254559_m1
*Fbxo32*		Mm00499523_m1
*Trim63*		Mm01185221_m1
*II6*		Mm00446190_m1
*TNFA*		Mm00443258_m1
*II1r1*		Mm00434237_m1
*Tgfbi*		Mm01337605_m1
*Rpl32*		Mm02528467_g1
*TJP1*	*Homo sapiens*	HS01551861
*CLDN1*		HS00221623 m1
*HSPA2*		Hs00745797_s1
*IL1B*		Hs01555410_m1
*CXCL8*		Hs00174103_m1
*EEF1A1*		Hs00265885_g1

### 2.5 Immunofluorescence

Tibialis anterior, TA, was collected from old mice (14 mo) fed a regular mouse diet supplemented with 0 and 8% ESM for 10 weeks and compared with young mice (3 mo) fed a regular mouse diet without ESM. TA muscles (*n* = 4) were embedded in OCT compound (Tissue Tek, Sakura Finetek, United States) and then snap-frozen in liquid nitrogen. Five micrometer-thick cryo-sections were cut on a cryostat and mounted on poly-L-lysine-coated glass slides. The sections were air-dried for 10 min, stained for 10 min with WGA Alexa Fluor^™^ 488 (Molecular Probes, Thermo Fisher Scientific) and Hoechst (33,342, Thermo Fisher Scientific), washed twice in PBS and mounted using ProLong^™^ Diamond Antifade Mountant (Thermo Fisher Scientific). The sections were examined by fluorescence microscopy analysis (apotome mode; ZEISS Axio Observer Z1 microscope, Germany), and images were processed using Adobe Photoshop CS3. If needed, brightness and contrast were adjusted manually across the entire picture. Myofiber cross-sectional area, minimum Feret diameter, centronucleated fibers, and number of fibers in the young and the old group TA muscles were quantified based on the WGA/Hoechst staining using MuscleJ plugin from ImageJ (30).

To quantify myofiber types, the sections were sequentially stained against Myosin heavy chain IIB on day 1, followed by Myosin heavy chain IIa/IIx on day 2. The cryo-sections were air-dried for 10 min, incubated with blocking buffer (5% BSA in PBS) for 1 h, and briefly washed in PBS before incubation with α-MCH IIb (BF-F3, DSHB, United States) overnight at 4°C, diluted in 1% BSA. Subsequent washing was performed three times with PBS before incubation with a secondary antibody (Alexa 555 mouse α-goat IgM, A21426, Thermo Fisher Scientific) diluted in 1% BSA for 1 h. The sections were rewashed and re-incubated in a blocking buffer for 30 min, washed briefly, and incubated with α-MCH IIa (SC-71, DSHB) diluted in 1% BSA for 90 min at room temperature. Sections were washed three times, incubated with secondary antibody (Alexa 488 Goat α-mouse IgG, A10667, Thermo Fisher Scientific) diluted in 1% BSA for 1 h, and finally washed three times and mounted using ProLong^™^ Diamond Antifade Mountant (Thermo Fisher Scientific). Myofiber types were quantified using the cell counter plugin from ImageJ.

### 2.6 Proteomics analysis

#### 2.6.1 Sample preparation for proteomics analysis

Approximately 50 mg TA muscles (*n* = 5) collected from Young, Old, 1% ESM, and 8% ESM mice were subjected to extraction of metabolic and cellular proteins by homogenization in 500 μL TES-buffer (10 mM Tris, pH 7.6, 1 mM EDTA, 0.25 M sucrose) using a Precellys24 (#74106, Bertin Technologies, France) at 6000 rpm for 2×20 s. The soluble proteins were further reduced with DTT, alkylated with IAA, and digested with Trypsin/Lys-C overnight (31). After sonication, peptides were purified and concentrated using StageTip, C18 material filled in 20 μL pipette tips (31) and (32), and finally dried completely with a speed-vac (Thermo Fisher Scientific, United States).

#### 2.6.2 Label-free liquid chromatography–tandem mass spectrometry and data analyses

The dried peptides (10 μg) were resolved with 10 μL resolving buffer (2% (v/v) ACN and 0.05% (v/v) trifluoroacetic Acid). From each peptide sample, one μg was loaded onto a trap column (Acclaim PepMap100, C18, five μm, 100 Å, 300 μm i.d. × 5 mm, Thermo Fisher Scientific), and backflushed with the loading buffer (2% (v/v) ACN and 0.05% (v/v) formic acid (FA)) onto a 50 cm × 75 μm analytical column (Acclaim PepMap RSLC C18, two μm, 100 Å, 75 μm i.d. × 50 cm, nanoViper, Thermo Fisher Scientific). All samples were analyzed with two technical replicates using a 2-h gradient elution profile. For detailed descriptions of the LC conditions and Q-Exactive Quadrupole-Orbitrap mass spectrometer (Thermo Fisher Scientific) settings, see (33). MaxQuant (Version 1.6.7.0) was used for label-free protein quantification analysis, with the following settings: carbamidomethylation (C) as fixed modifications; oxidation (M), N-terminal acetylation and deamidation (NQ) as variable modifications; a maximum of two missed cleavages; a precursor peptide tolerance of 10 ppm; a fragment mass tolerance, 0.020 Da; minimum two peptide matches. Only proteins quantified in at least one sample per group were included in the following analyses (954 proteins).

### 2.7 Microbiota analysis

Cecum samples were shipped to ZIEL Core Facility Microbiome (TUM, Germany) and analyzed for microbiota composition following the procedure described by (35, 36). Briefly, following DNA extraction, the variable region V3-V4 of the 16S rRNA gene was amplified in duplicates per sample and sequenced in a paired-end mode on an Illumina MiSeq (37). The sequencing data was preprocessed using the IMNGS and Rhea pipelines (38, 39). IMNGS is based on the UPARSE approach (40), and involve sequence quality check, chimera filtering using UCHIME (40, 41), cluster formation into Operational Taxonomic Units (OTUs) using USEARCH v11.0 (41), removal of non-16S sequences using SortMeRNA v4.2 (42) and taxonomic annotation by SINA v1.6.12012 (43) using SILVA release 128 as reference. OTUs occurring at a relative abundance <0.25% across all samples were removed to prevent the analysis of spurious OTUs (35). Using Rhea scripts (available from)^1^ in an R programming environment, OTU tables were normalized to account for differences in sequence depth by division to their sample size and then multiplication by the size of the smaller sample. Furthermore, microbiota (alpha) diversity analysis included different indices or their effective numbers (Richness, Shannon, Simpson, Evenness) and taxonomic binning was performed as explained in detail in Rhea.

### 2.8 *In vitro* digestion of ESM-powder, ESM-Gag, and ESM-peptide fraction

Samples were subjected to a static *in vitro* digestion model simulating the oral-, gastric- and intestinal phases. The model was based on the INFOGEST digestion protocol with standardized electrolyte solutions for the preparation of simulated salivary fluid (SSF), gastric fluid (SGF), and intestinal fluid (SIF) (44). To simulate the digestive process in adults, 250 mg of sample were mixed with 750 mg water, 1 mL of SSF containing salivary amylase (50 U/mL) and kept at 37°C during shaking for 2 min. The gastric phase was simulated by adding 2.0 mL of SGF-containing pepsin (P7000, Sigma-Aldrich Co, United States), resulting in a pepsin activity of 2000 U/mL in the final volume. The pH was adjusted to 3.0 before incubation in a rotary incubator (Innova^®^ 40/40R, New Brunswick Scientific, Edison, NJ, United States) at 37°C and 215 rpm for 120 min. To simulate the intestinal phase, samples were added 4 mL of SIF containing 0.07 mM NaHCO_3_, porcine bile (B8381, Sigma-Aldrich Co, United States), and pancreatin (P1750, Sigma-Aldrich Co, United States), resulting in a bile salt concentration of 10 mM and pancreatin concentration of 1.25 mg/mL in the final volume. After adjustment to pH 7.0, the samples were further incubated at 37°C and 215 rpm. The present pancreatin concentration results in a last trypsin activity of 10 U/mL in the intestinal phase, less than INFOGEST recommended (100 U/mL). However, the lower pancreatin concentration had only a minor impact on *in vitro* protein digestibility (unpublished results). In contrast, high amounts of background protein could be avoided, making interpreting results easier. A similar digestion model was used to mimic the digestive process in older adults based on literature stating that aging is accompanied by changes in secretion and composition of saliva, less gastric fluid (higher gastric pH and reduced pepsin levels), and lowered bile and reduced levels of pancreatic enzymes (45). In the present project, the digestive process in older adults was simulated by lowering the level of pepsin in the gastric phase to 500 U/mL while increasing the pH to 4.5 and lowering the concentrations of bile and pancreatin resulting in final concentrations of 5 mM bile and 4 U/mL trypsin activity in intestinal phase. All *in vitro* digestion experiments were performed in triplicates. Blank samples (water) were included in the experiments to estimate the contribution from digestive fluids, and all measurements were corrected for the background.

To determine protein digestibility, tubes were withdrawn after 80 min in the intestinal phase and heat treated at 90°C for 10 min to stop the enzyme activity. After that, the samples were centrifuged at 4000 rpm for 10 min, and aliquots of the supernatant were stored at minus 20°C until further analysis. As previously described, the peptide size distribution of supernatants was determined using size exclusion chromatography (SEC) (46, 47). Before injection, the samples were filtered using Millex-HV PVDF 0.45 μm 33 mm filters (Merck), and the injection volume was 10 μL. The HPLC system consisted of a pump (Dionex UltiMate 3,000), an auto-injector (Dionex UltiMate 3,000), a chromatographic column (BioSep-SEC-s2000, Phenomenex, 300–7.8 mm) kept at room temperature, and a UV detector at 214 nm. A mixture of acetonitrile (30% v/v) and ultrapure water (70% v/v) containing 0.05% trifluoracetic acid (TFA) was used as eluent with a flow rate of 0.9 mL/min. Isocratic separation was carried out for 17 min. Between 17 and 20 min, 100 mM NaH2PO4 was used as the mobile phase for column cleaning. After 20 min, the mobile phase was switched back to 30% acetonitrile, and the column was equilibrated for another 30 min. Chromatographic runs were controlled using Chromeleon software. Each chromatogram was divided into two parts: (1) retention times from 5.0 to 9.4 min (larger peptides and proteins) and (2) retention times from 9.4 to 15.0 min (smaller peptides and amino acids). Excel was used to estimate the total area under the curve (SEC area), reflecting the amounts of soluble proteins and the percentage of small peptides (% small peptides) reflecting the degree of hydrolysis.

### 2.9 Cell cultures and treatments

Immune-modulating properties of ESM powder were analyzed as in the previous study (18) by use of the U937-3xκB-LUC cells and the human monocytic leukemia cell line THP-1 (purchased commercially; TIB-202TM; ATCC, United Kingdom) in addition to Caco2 cells and primary bovine skeletal muscle cells described below. The immune cells were susceptible to the digestion-solution of the *in vitro* system and, therefore, were not subjected to samples after *in vitro* digestion as for the skeletal muscle analysis. In brief, the U937-3xκB-LUC cells, a human monocytic cell line stably transfected with a luciferase reporter containing 3 x NF-κB binding sites, were maintained in RPMI-1640 medium supplemented with 10% FBS, two mM L-glutamine, 50 U/mL penicillin, 50 mg/mL streptomycin and 75 μg/mL hygromycin. All experiments with U937 cells were performed in a medium with 2% FBS. To measure NF-κB activity, cells were seeded into white 96-well plates with a density of 1 ×10^5^ cells/ml, 100 μL/well, and incubated with either only one μg/mL LPS (*Escherichia coli* 055:B5; Sigma-Aldrich, United States) or in the presence of various concentrations of ESM powder for 6 h. The NF-κB activity was determined by measuring the luciferase activity after the addition of Bright-Glo Reagent (Promega, Madison, WI) by the manufacturer’s instructions. Luminescence was detected using a Glomax96 Microplate Luminometer (Promega). THP-1 was grown in suspension in complete RPMI 1640 culture medium supplemented with 10% fetal bovine serum (FBS), 100 U/mL penicillin, 100 μg/mL streptomycin, two mM L-glutamine and 0.05 mM β-mercaptoethanol. The mature macrophage-like state of THP-1 cells was induced by treating the cells (10^6^ cells/ml) for 72 h with 100 ng/mL of phorbol 12-myristate 13-acetate (PMA; Sigma-Aldrich, United States) in 12-wells culture plates. Differentiated, plastic-adherent cells were washed twice with sterile Dulbecco’s phosphate-buffered saline (Sigma-Aldrich) and then incubated with fresh medium without PMA. Experiments were performed on the second day after PMA removal. Differentiated cells were treated with LPS (0.5 ng/mL) alone or with LPS plus 0.5 mg/mL and 1 mg/mL ESM for 20 h. The media was collected, centrifuged to remove cell debris, and subjected to ELISA for measuring cytokines secretion.

The human colorectal adenocarcinoma cell line Caco-2 (HTB-37) was obtained from the American Type Culture Collection, grown in Dulbecco’s modified Eagle’s medium supplemented with 20% heat-inactivated FBS, 1% non-essential amino acids, 100 U penicillin ml − 1 and 100 μg streptomycin ml − 1. The cells were maintained at 37°C in a humidified atmosphere of 5% CO2 and subcultivated at 70–90% confluence. To obtain polarized monolayers, Caco-2 cells were seeded onto cell culture inserts (0.4 μm pore size, 12 mm diameter, polyethylene terephthalate, Millipore Bedford, MA) at a concentration of 3 × 10^5^ cells per filter for 5 weeks. The filters were maintained with a volume of 1 mL in the apical compartment and 2 mL in the basolateral chamber, and cell media was changed three times per week. Differentiation of the cells was checked by measurement of transepithelial electrical resistance (TEER) with a Millicell-ERS electrode (Millipore). TEER values were more than 500 Ω cm_2_ after 4 weeks of incubation. For transport experiments, cells were washed twice with sterile PBS, and serum-free DMEM replaced the medium without phenol red (1.3 mL basolateral). Serum-free DMEM was added to the digested ESM fractions to reach 1% and incubated for 24 h before analysis. Fluorescein-dextran 10 kDa (Sigma Aldrich, St. Louis, MO) was added to the apical side of each filter at a concentration of 1.85 mg/mL as a control for unspecific transport across the Caco-2 monolayer. Aliquots of two times 25 μL of the basolateral medium were assayed for fluorescein content in a microplate fluorescence spectrophotometer (Fluorstar Optima, BMG Labtech, Ortenberg, Germany) and two times 500 μL of the basolateral medium were stored frozen for calcofluor fluorescence measurements. If not otherwise stated, all cell culture solutions were obtained from Invitrogen (Carlsbad, CA).

Skeletal muscle regeneration and immune-modulating properties of *in vitro* digested ESM powder and carbohydrate-rich fraction were evaluated using primary bovine skeletal muscle cells (MuSCs). MuSCs were isolated essentially as described (48, 49). In brief, the isolated bovine skeletal muscle cells were sub-cultivated in proliferation media consisting of DMEM containing 10% FBS, 2% Ultroser, P/S (10 000 units / mL) and Amphotericin B (250 μg/mL) in 75 cm2 Entactin-Collagen-Laminin cell attachment matrix (ECL; Merck, Kenilworth, NJ, United States)-coated culture flasks before cell experiments. All experiments were performed in 2nd or 3rd passage. Proliferation experiments were performed in 96 well ECL-coated plates seeded out onto black (cell proliferation assays) or white opaque (cell viability assays) microtiter plates at a cell density of 3000 cells/well in a proliferation medium and incubated for approximately 24 h. Samples dissolved in proliferation medium, or only medium (control), were added to cells and incubated for 2 days before cell proliferation and viability analysis. Immune modulating properties were determined by seeding 5.000 cells/cm^2^ in 6 well ECL-coated plates. Confluent skeletal muscle cells were then stimulated with 10 ng/μl TNFA for 2 days in a differentiation medium consisting of DMEM containing 10% FBS, 2% Ultroser, P/S (10 000 units / mL), and Amphotericin B (250 μg/mL) added 1% *in vitro* digested samples or not (control). Cells were then washed 2x with PBS and harvested for RNA analysis according to the manufacturer’s protocol of the RNeasy mini kit or RIPA buffer for western-blot analysis. Cell proliferation was measured using the CyQUANT cell proliferation assay kit (ThermoFisher Scientific) according to the manufacturer’s instructions. The fluorescence and intensity of cell proliferation was detected using the FLUOstar OPTIMA microplate reader (BMG LABTECH GmbH, Ortenburg, Germany).

### 2.10 Human small-scale preliminary study

The study was conducted at the University of Oslo, Norway, from October 2020 until April 2021. The study was designed as a 4-week randomized controlled parallel double-blinded trial. It was considered a small-scale preliminary study with aim to evaluate oral supplementation of ESM as an anti-inflammatory ingredient in older subjects. Participants were recruited through advertisements on social media. After a telephone interview, we invited eligible participants to a baseline visit. Participants were included if they were home-dwelling men and women ≥70 years. They were excluded if they had CRP >10 mg/L, had diabetes type I or II, HbA1c ≥ 6.5%, had an egg allergy, used medication affecting inflammation, had unstable use of drugs or supplements last 3 months, were unwilling to keep the physical activity level, habitual dietary intake, tobacco use, and weight stable during the study period, had severe illness last 3 months, and were unwilling to perform physical tests.

The intervention consisted of one intervention and one placebo group. Both groups were instructed to take two capsules with breakfast daily for 4 weeks (study end). The daily dose of ESM was equal to other previous human studies using ESM as hydrolysate (500 mg/day). Participants in the placebo group were given a supplement without ESM (cellulose, an indigestible carbohydrate). The ESM was produced at the Norilias/Norturas production center at Revetal, Norway, and washed and milled to a powder as described in the section below. The Norwegian Food Safety Authority (Mattilsynet) in Norway has approved this production site for the production of ESM for human consumption.

Subjects were stratified by gender and smoking and randomly allocated to either of two groups by an external statistician. The participants and researchers involved in the study were blinded to group allocation. In total, 123 subjects were assessed for eligibility, 57 were randomized, and 28 and 29 were allocated to either placebo or intervention. In total, 19 in each group were analyzed. All participants were advised to maintain their usual lifestyle habits throughout the study without changing their physical activity and dietary habits.

Blood samples were taken at baseline and end of intervention. All blood samples were analyzed at Fürst, a commercial laboratory in Oslo. Standard biochemical markers such as total cholesterol, LDL-C, HDL-C, Apo A1, Apo B, Lp(a), glucose, insulin, HbA1c, hs CRP, ASAT, ALAT, Creatinine, and eGFR were measured. The study was conducted according to the guidelines in the Declaration of Helsinki. All participants gave written informed consent, and the Regional Ethics Committee for Medical Research in Southeast Norway approved the study. The study was registered at ClinicalTrials.gov (ClinicalTrials.gov Identifier: NCTNCT04606628).

### 2.11 Cytokine protein analysis in cell experiment and blood samples (by ELISA)

#### 2.11.1 Cell culture

Levels of TNFA, ILB, IL6, IL8, and IL10 in cell culture supernatants were determined using an enzyme-linked immunosorbent assay (ELISA). MaxiSorp ELISA plates (Nunc, Roskilde, Denmark) were pre-coated with mouse anti-human IL1B (R&D Systems, Minneapolis, United States), rat anti-human IL6, IL8 or IL10 antibodies (BD Biosciences, San Jose, CA) and rat anti-human TNFA (BD Biosciences, San Jose, CA) suspended in coating buffer (0.1 M carbonate/bicarbonate buffer pH 9.6) overnight at 4°C. Unspecific binding sites were blocked by incubating with 5% BSA in PBS for 1 h at RT. Samples and human recombinant IL1B, IL10 (R&D Systems), or IL6 (BD Biosciences) standards diluted in high-performance ELISA (HPE) buffer (Sanquin, Amsterdam, Netherlands) were added. The plates were incubated for 1.5 h at RT before the biotinylated goat anti-human IL1B (R&D Systems), rat anti-human IL6, IL8 or IL10 (BD Biosciences), and rat-anti-human TNFA antibodies were added and further incubated for 1 h. Next, streptavidin-horseradish peroxidase conjugate (BD Biosciences) in HPE buffer was added and incubated at RT for 30 min. The plates were washed thrice with PBS containing 0.01% Tween-20 between these steps. After the last washing step, TMB single solution (Life Technologies, United States) was added and incubated for 10 min in the dark at RT. The reaction was stopped by adding 1 N H_2_SO_4_, and absorbance was measured at 450 nm using Titertek Multiscan plus MK II plate reader (Labsystems, Helsinki, Finland).

#### 2.11.2 Mice serum

Levels of TNFA in serum samples from mice were measured using ProcartaPlex, mouse five plex (ThermoFisher, Vienna, AUT) in a Bio-Plex 200 instrument (Bio-Rad, Hercules, CA, United States) and analyzed according to the manufacturer’s protocol. The samples were measured in duplicates.

#### 2.11.3 Human study

High sensitivity TNFA and CRP were analyzed using Quantikine ELISA (R & D Systems Inc. Minneapolis, United States) by the protocols provided. The interindividual variation (CV) was 10.5%, and all samples were measured in duplicates.

### 2.12 Statistical analyses

Gene expression and ELISA in cell studies: Results were expressed as mean ± standard error mean (SEM) of at least three independent cell seeding experiments performed in technical triplicates. Significant differences between treatments and the control sample were determined by one-way ANOVA using Dunnett’s multiple comparison test. Differences were considered significant at *p* < 0.05. Gene expression, histology and physiological tests in mice trial: All results from the mice trial were expressed as mean ± standard error mean (SEM), and significant variance compared to control was calculated by Brown-Forsythe and Welch ANOVA using Dunnett’s multiple comparison test. All univariate data were statistically analyzed in Graph Pad Prism version 7.03 (GraphPadSoftware, La Jolla, CA, United States). Proteomics in mice trial: The LC–MS/MS data were log10-transformed and imported into Unscrambler^®^ X, version 10.4.1 (CAMO A/S, Oslo, Norway). Partial least squares regression was used to assess correlations between protein abundances and treatment groups. All variables were weighted (1/standard deviation), and full cross-validation was used to select optimal number of model components and for jack-knife uncertainty testing. Microbiota in mice trial: Relative abundances at phylum and genus level were analyzed in MATLAB (R2022b, The MathWorks Inc.) using multivariate statistical methods: 55 multivariate analysis of variance (FFMANOVA) (50) and ANOVA-simultaneous component analysis (ASCA) (51) both highly suitable for microbiome analysis (52). FFMANOVA was used to obtain *p*-values at both community and taxonomic levels. In contrast, ASCA was used to visualize the variation between mice groups (ASCA scores) and the bacterial taxa contributing to the variation (ASCA loadings). Significant differences in microbiota diversity between treatments and the old control sample were determined by one-way ANOVA using Dunnett’s multiple comparison test. Association study was performed by PLS regression with full cross validation for microbiota (at the genus level) and the most important hallmarks of ageing and inflammatory markers. Human study: The change in outcomes from baseline to end of study was calculated in the human study. We used a linear regression model to test if there was a difference in the change between ESM and placebo. For hsCRP, we also ran a model adjusted for the baseline concentration of hsCRP. *p* value<0.05 was considered significant. All analyses were performed in R.

## 3 Results

### 3.1 ESM intake in mice has no effects on coordination (rotarod), but an attenuating effect on loss of skeletal muscle grip strength after 4 weeks and a modest effect on body weight

**Figure 1:**
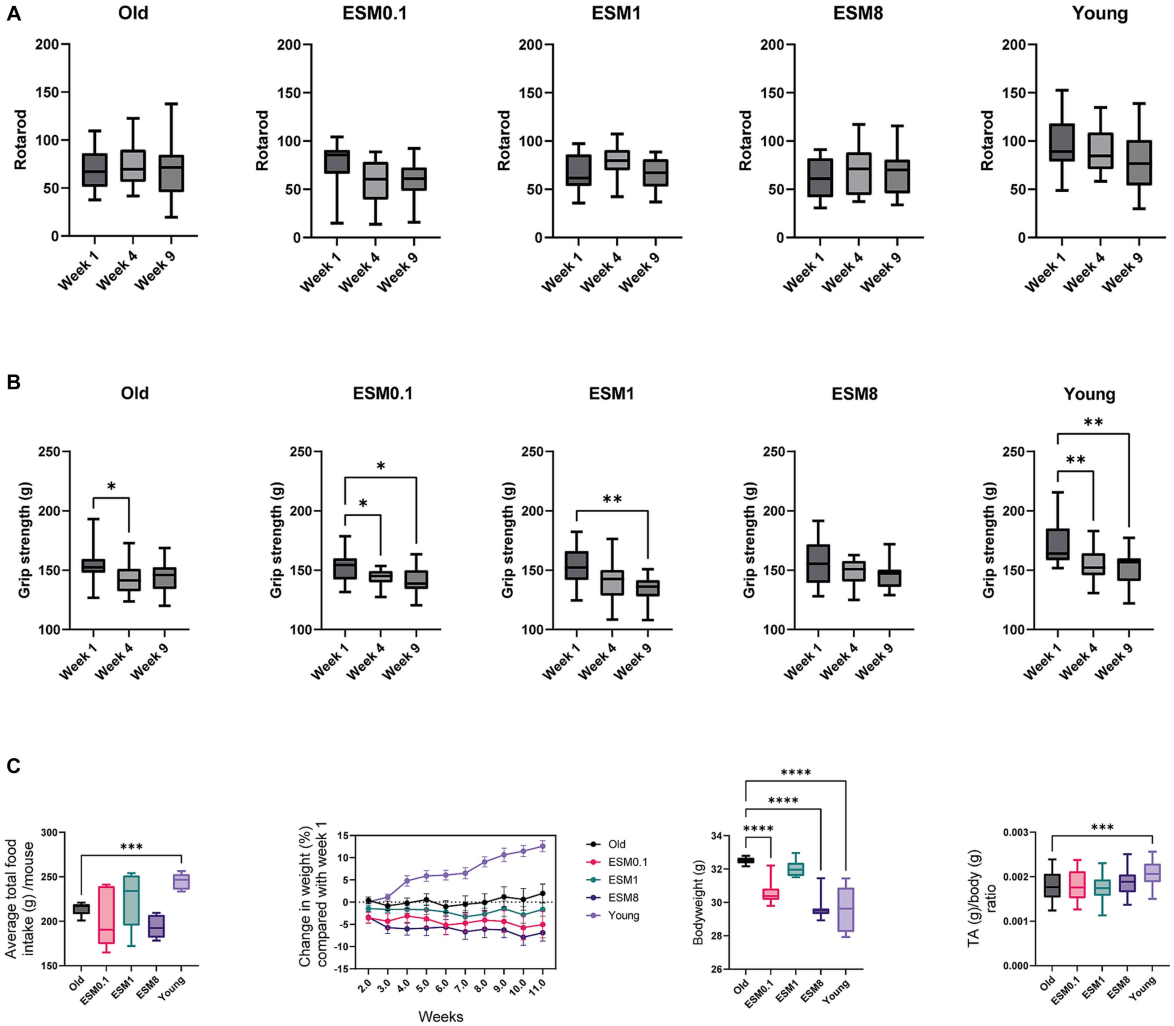
Old mice fed with 8% ESM supplemented diet maintain muscle strength. **(A)** Results from Rotarod tests done at weeks 1, 4, and 9. Rotarod tests were repeated six times over 2 days (three tests/day). **(B)** Results from Grip strength tests in week 1, 4 and 9 from all the experimental groups. All groups except ESM8 had reduced grip strength throughout the study. Values from each mouse are a mean of three grip tests in duplicate. **(C)** Left panel shows average total food intake per group over 10 weeks, the following two panels show body weight changes through the 10-week study (% relative to week 1) and body weight after 10 weeks, respectively. The right panel shows the TA/body weight ratio. Asterisks indicate significant differences compared to old mice, determined by Brown-Forsythe and Welch ANOVA using Dunnett’s multiple comparison test, *p < 0.5, **p < 0.01, ***p < 0.001, ****p < 0.0001.

The rotarod and grip tests were used to analyze aging phenotypes during the mouse experiment in old mice (14 months) fed a standardized AIN93 mouse diet supplemented with 0, 0.1, 1 and 8% ESM for 10 weeks, and compared with young mice (3 months) fed without ESM. We did not observe any changes in rotarod measurements obtained at the same time points regardless of ESM supplementation, indicating no effects of ESM on coordination (Figure 1A). Grip strength was measured at 1, 4, and 9 weeks (Figure 1B). In the old group without ESM supplementation and young group, a significant reduction in grip strength was observed after 4 weeks compared to 1 week. This reduction in grip strength was not observed in the old mice group supplemented 1 and 8% ESM at 4 weeks. A sustained attenuating effect on the decrease in grip strength was obtained in both the old mice and the 8% ESM group at 9 weeks. No significant difference between the groups was observed at the end of trial (data not shown). The average total food intake was lower for old mice compared to young mice, but no difference in total food intake was observed for old mice fed with ESM compared to the old control group (Figure 1C left). There were only minor changes observed in body weight (%) for all groups of old mice at week 11 compared to week 1, except for old mice fed with 8% ESM supplementation which showed a tendency for reduced bodyweight (Figure 1C middle left). Young mice showed a steady increase in bodyweight from week 1 to week 11. At the end of the experiment, the body weight of old mice with no ESM supplementation was significantly higher than aged mice fed with 0.1 and 8% ESM supplementation, and also significantly higher than young mice (Figure 1C middle right). No statistical changes were observed between old mice with no ESM compared to old mice fed with 1% ESM. Furthermore, Tibialis anterior (TA) and gastrocnemius (GTN) skeletal muscle mass measurements normalized to body weight demonstrated no difference between the groups of old mice (Figure 1C right TA and Supplementary Figure S1 GTN), however, there was a significant increase in TA/bodyweight in young mice compared to old mice.

### 3.2 ESM supplemented diet attenuates morphological and molecular hallmarks of aging in mouse skeletal muscle

**Figure 2:**
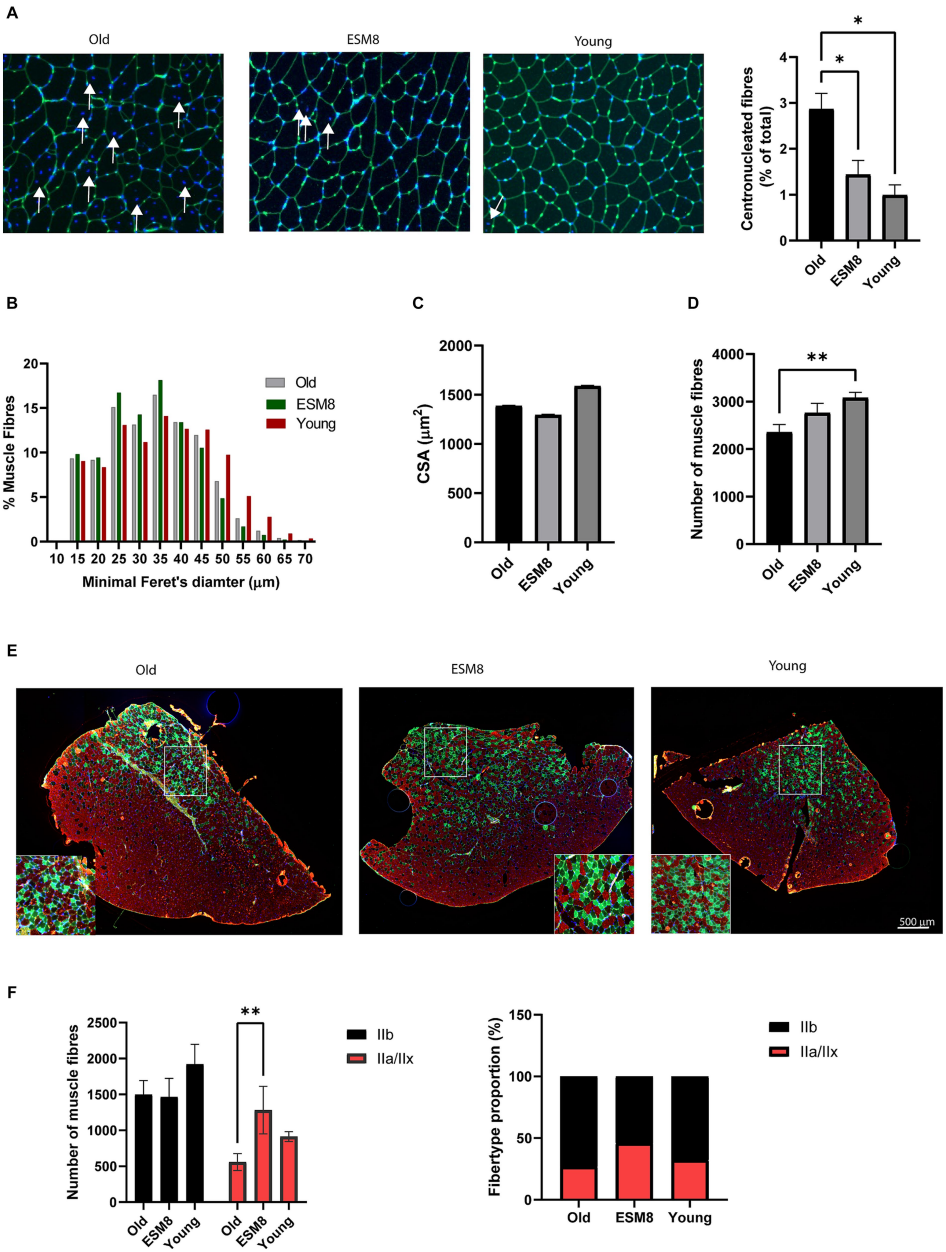
Muscle fiber type and fiber-type composition in old mice with fed with 8% ESM supplement resembled young mice. **(A)** Representative images of the lectins in the connective tissue of TA from the different groups stained with WGA (green). The nuclei were counterstained with Hoechst (blue). Arrows indicate displacement of the nucleus in skeletal muscle fibers (left). Bars (right) show the quantification of centronucleated fibers in pictures (*n* = 3–5) from each group using the ImageJ MuscleJ plug-in. **(B)** Bars show minimum Feret’s diameters, **(C)** average CSA, and **(D)** number of muscle fibers in the three groups analyzed using the Image J MuscleJ plugin. *n* = 1800–3,700 fibers/image; 9–10 images/condition. **(E)** Representative immunofluorescence picture of TA muscle stained for type IIa (SC-71 antibody, green) and type IIB (BF-F3, red). Nuclei were counterstained using Hoechst (blue). Scalebar as indicated. **(F)** Bars show numbers of fibers (left) and fiber type proportion (right), quantified using ImageJ. *n* = 4 animals. Asterisks indicate significant differences compared to old mice, determined by Brown-Forsythe and Welch ANOVA using Dunnett’s multiple comparison test, *p < 0.5, **p < 0.01.

Histological analysis of TA muscle from old mice revealed a substantially higher number of centrally nucleated fibers compared to muscle from young mice (Figure 2A), indicating extensive muscle degeneration/regeneration (53, 54). The appearance of increased centronucleated fibers in old mice was highly reduced after intake of 8% ESM. We further determined whether ESM intake also affected skeletal muscle fiber size. The cross-section area of TA from older mice had an average minimal Feret’s diameter fiber size distribution pattern shifting to the left, demonstrating the presence of smaller size fibers in older mice (Figure 2B). There was no difference in the cross-sectional area (CSA) comparing the groups, although old mice had slightly lower CSA (Figure 2C). The total number of fibers per TA muscle was significantly reduced in old mice versus young mice (Figure 2D), indicating a significant loss of muscle fibers with age. Loss of muscle fibers were less evident in old mice fed with 8% ESM diet compared to young mice To define whether ESM intake affected fiber type specificity in muscle atrophy, TA cross sections were immunolabeled for myosin heavy chain (MHC) isoforms (Figures 2E,F). Compared to young mice, old mice exhibited a discernible reduction in type IIb and type IIa/IIx muscle fibers, reflecting atrophy in skeletal muscle fiber types during aging. Intake of ESM attenuated the decrease in type IIa/IIx fiber type in old mice. Histological staining of extracellular matrix in skeletal muscle of old mice with and without 8% ESM supplemented diet revealed no visible fibrosis (Supplementary Figure S2A). At the gene level, gene expression of the fibrosis markers Collagen alpha-2(I) chain (*Col1a2*) and Collagen alpha-1 (III) chain (*Col3a1*) was reduced in old mice compared to young mice. Also, we did see an increase in gene expression of Transforming growth factor beta-1 proprotein (*Tgfbi*) and *Col1a2* expression with 8% ESM in the diet compared to old mice without ESM supplement in the diet (Supplementary Figure S2B). No change in the relative gene expression of the inflammatory markers Interleukin-6 (*Il6*) and Interlukin-1 receptor (*Il1r*; Supplementary Figure S2C) was observed in old mice when ESM was supplemented in the diet. However, we did see a higher gene expression of *Il1r* in the young mice compared to old mice.

**Figure 3:**
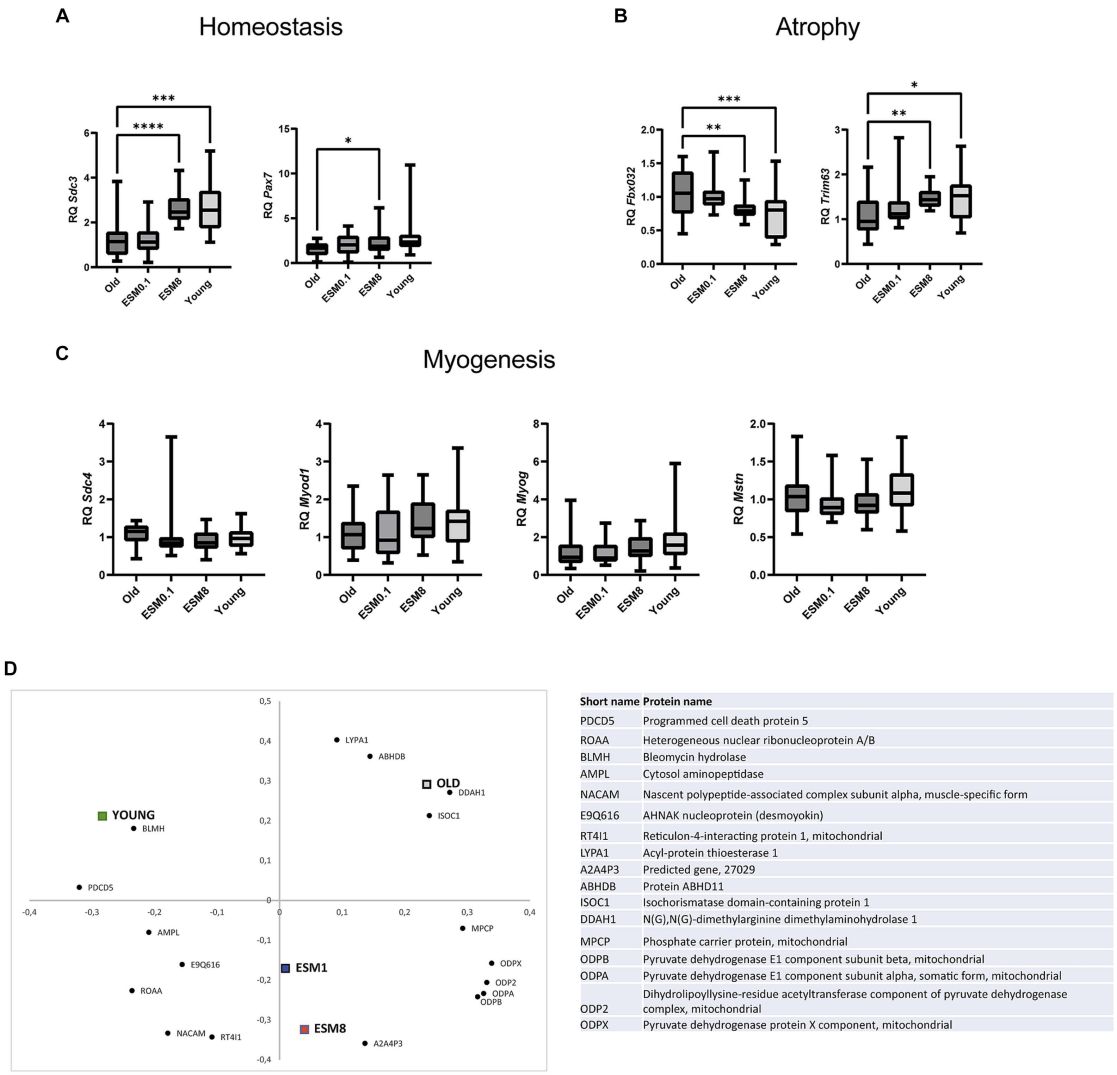
ESM supplement restores skeletal muscle homeostasis and atrophy but not myogenesis in the mouse TA muscle. Bars show the relative gene expression of **(A)** homeostasis markers *Sdc3* and *Pax7*, **(B)** atrophy markers *Fbxo32* and *Trim63*, and **(C)** myogenesis markers *Sdc4*, *Myod1*, *Myog* and *Mstn* in old mice with or without ESM. Asterisks indicate significant differences compared to old mice, determined by Brown-Forsythe and Welch ANOVA using Dunnett’s multiple comparison test, *p < 0.5, **p < 0.01, ***p < 0.001, ****p < 0.0001. **(D)** Loading plot from the partial least squares (PLS) regression analysis of LC-MS/MS data from the sample groups showing the first two principal components, explaining 26 and 21% of the variation in the data set, respectively. All proteins included in the figure contribute significantly toward grouping the different treatment groups, and protein names are given in the table (right).

Gene expression of genes related to skeletal muscle atrophy, regeneration og myogenesis were then examined in TA (Figure 3) and GTN (Supplementary Figure S3). This assessment was performed considering histological findings that suggested disparities in regeneration, characterized by centrally nucleated fibers and atrophy, marked by the loss of muscle fibers. Relative gene expression of the homeostasis marker Syndecan-3 (*Sdc3*) was reduced in muscles of old mice compared to the young mice (Figure 3A). Intake of 8% ESM significantly restored the expression levels of this marker to the levels found in the young mice (Figure 3A). Intake of 8% ESM also increased the gene expression of the satellite cell marker Paired box protein Pax-7 (*Pax7*). Likewise, intake of 8% ESM also restored the atrophy markers F-box only protein 32 (*Fbxo32*) and E3 ubiquitin-protein ligase TRIM63 (*Trim63*) to the levels observed in young mice (Figure 3B). No differences were observed in mRNA expression of the myogenesis markers Syndecan-4 (*Sdc4*), Myoblast determination protein 1 (*Myod1*), Myogenin (*Myog*), and Myostatin (*Mstn*) between the different groups (Figure 3C). Interestingly, the gene expression pattern was different in GTN muscle, where differentiation markers for myogenesis were reduced in old mice compared to young mice and affected by ESM supplementation (Supplementary Figure S3). Likewise, markers for atrophy and homeostasis were unaffected by age, strongly indicating skeletal muscle differences.

Skeletal muscle undergoes a shift to a more aerobic-oxidative metabolism in lower-twitching fiber population upon aging, and we therefore investigated the water-soluble protein fraction extracted (proteins involved in cellular processes and metabolism) from old mice with and without ESM supplementation and young mice by proteomics analysis. The proteomics data’s partial least squares (PLS) regression analysis revealed systematic differences in protein abundance between the groups, with 17 proteins significantly contributing to the separation along the first two principal components (Figure 3D). The old control mice and young mice were separated along the first principal component (PC), with the old mice fed 1 and 8% ESM in the center, while the second PC separated the two ESM groups from the old and young groups. These findings indicate that changes are occurring with the old mice supplemented with ESM, leading to a separation from the old control mice and potentially reflecting a shift toward a protein profile more like the young mice. Proteins associated with this shift (i.e., located in the proximity of the ESM groups in the loading plot) were related to energy metabolism (mitochondrial pyruvate dehydrogenase isoforms), protein synthesis (amino acid AA-synthesis carrier protein A2A4P3), and myotube development (Nascent polypeptide-associated complex subunit alpha, muscle-specific form, NACAM).

### 3.3 Effect of ESM supplementation on inflammatory markers

**Figure 4:**
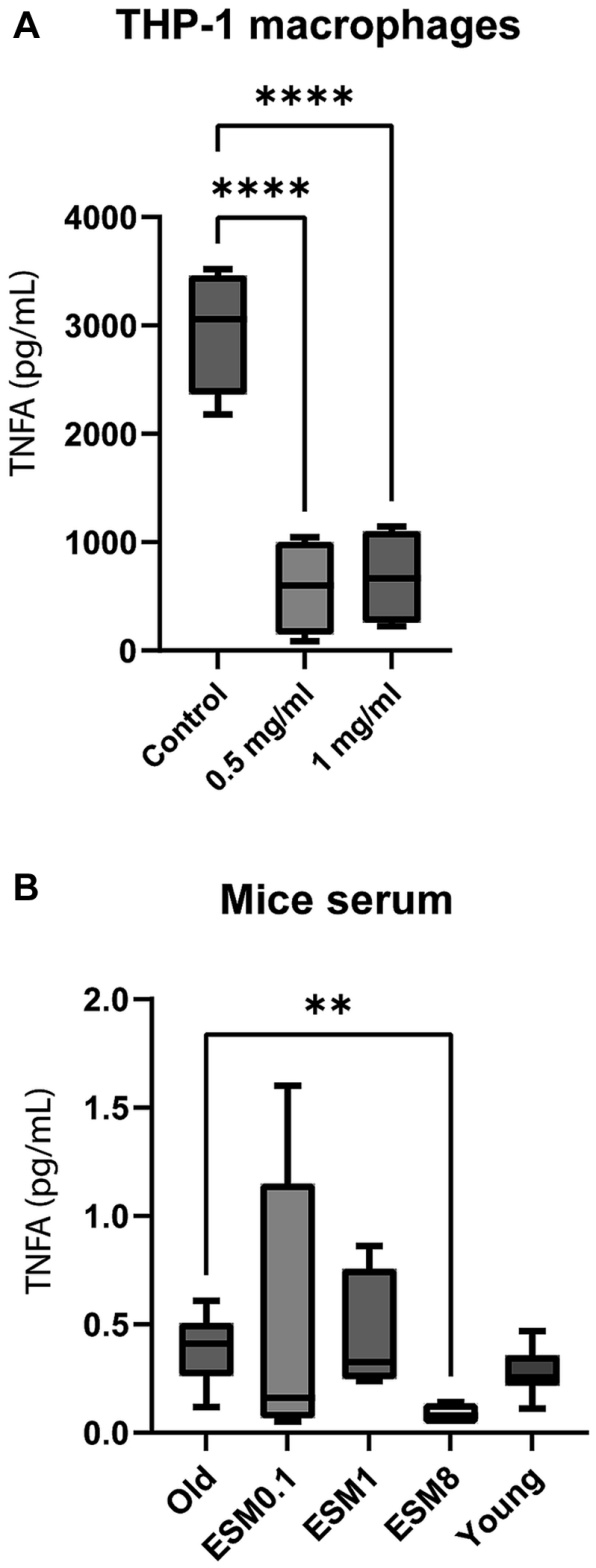
ESM reduces the level of TNFA *in vitro* (cells) and *in vivo (mice)*. **(A)** PMA-differentiated THP-1 cells were incubated with 0.5 ng/mL LPS alone (control) or in combination with either 0.5 or 1 mg/mL ESM for 20 h at 37°C. The cell media were collected, and ELISA determined the level of TNFA. The results are presented as mean ± SEM protein level (*n* = 3). Asterisks indicate significant difference, ****p < 0.0001 between control and ESM treated cells, determined by unpaired t-test. **(B)** Serum samples collected from old mice fed with 0, 0.1, 1, and 8% ESM supplemented feed for 10 weeks and young mice fed without ESM were determined for TNFA level by Multiplex ELISA. The results are presented as mean ± SEM of all mice within each group (*n* = 15). Asterisks indicate significant differences compared to old mice determined by Brown-Forsythe and Welch ANOVA using Dunnett’s multiple comparison test, **p < 0.01.

**Figure 5:**
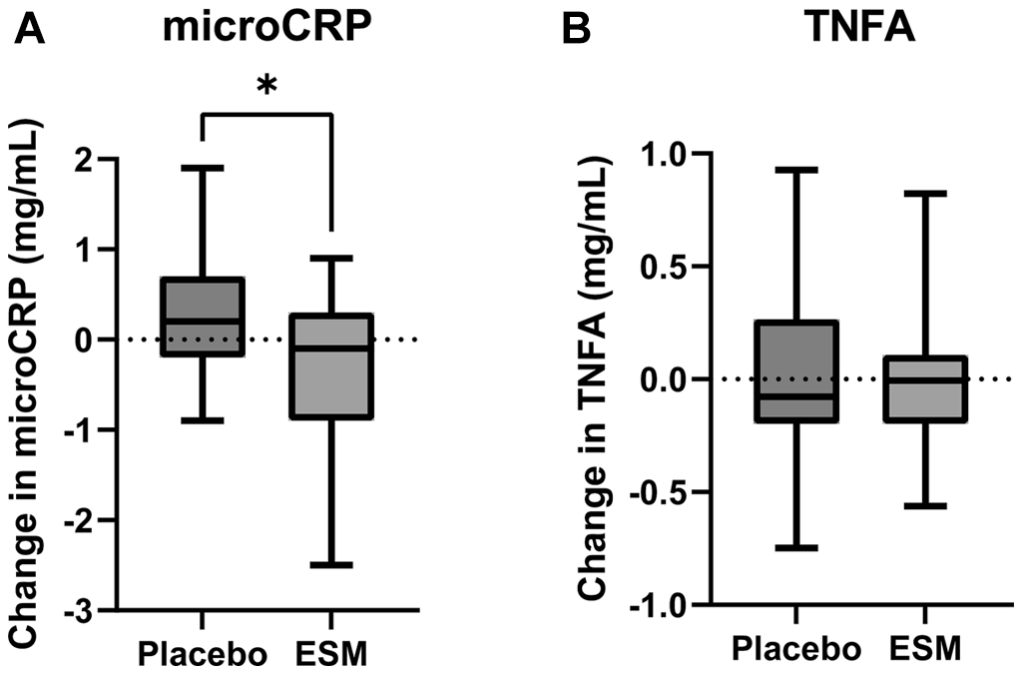
Intake of ESM reduces hsCRP in healthy home-dwelling elderly. Change in hsCRP **(A)** and hsTNFA **(B)** from baseline to end of study (4 weeks) in placebo and ESM group, measured with ELISA. Asterisks indicate significant differences between groups. The results are presented as mean ± SEM, and each point represents individual values. To test if there was a difference in the change between ESM and placebo a linear regression model was used. *p* value * < 0.05 was considered significant.

We investigated a possible immune modulating effect of ESM on low-grade inflammation, both *in vitro* using THP-1 macrophage cells (Figure 4A; Supplementary Figure S4A), *in vivo* by analyzing serum from mice (Figure 4B; Supplementary Figure S4B) and serum from humans collected in a randomized controlled small-scale preliminary study with elderly (≥70 years) receiving 500 mg ESM per day (Figure 5).

Tumor necrosis factor (TNFA) was significantly reduced in the LPS-stimulated THP-1 macrophages supplemented with ESM compared to the control (Figure 4A). Immune modulating effect on cytokines (IL1B, IL6, IL10), as previously shown (18), was confirmed in our study (18) using *in vitro* in THP-1 macrophages (Supplementary Figure S4A). The TNFA level decreased in the serum of old mice fed with 8% ESM compared to old mice with no ESM in the diet (Figure 4B). However, there were no significant differences in IL2, IFG, and IL6 serum levels between the different groups (Supplementary Figure S4B).

In the small-scale preliminary human study with healthy home-dwelling older adults (*n* = 38), there were 21% men in the placebo group and 16% men in the ESM group. In both groups, the fasting serum glucose concentration and total cholesterol, LDL and HDL cholesterol were within the normal ranges. The baseline concentration of hsCRP was 1.4 mg/L in placebo and 1.7 mg/L in ECM (Supplementary Table 1). The concentration of hsCRP was significantly reduced in the group receiving 500 mg/day ESM for 4 weeks compared to the placebo group (Figure 5A). This difference remained significant after adjusting for baseline CRP levels. However, neither the concentration of hsTNFA (Figure 5B) nor any of the secondary muscle functions measured were altered during the intervention (Supplementary Table 2).

### 3.4 *In vitro* protein digestibility of micronized ESM

**Figure 6:**
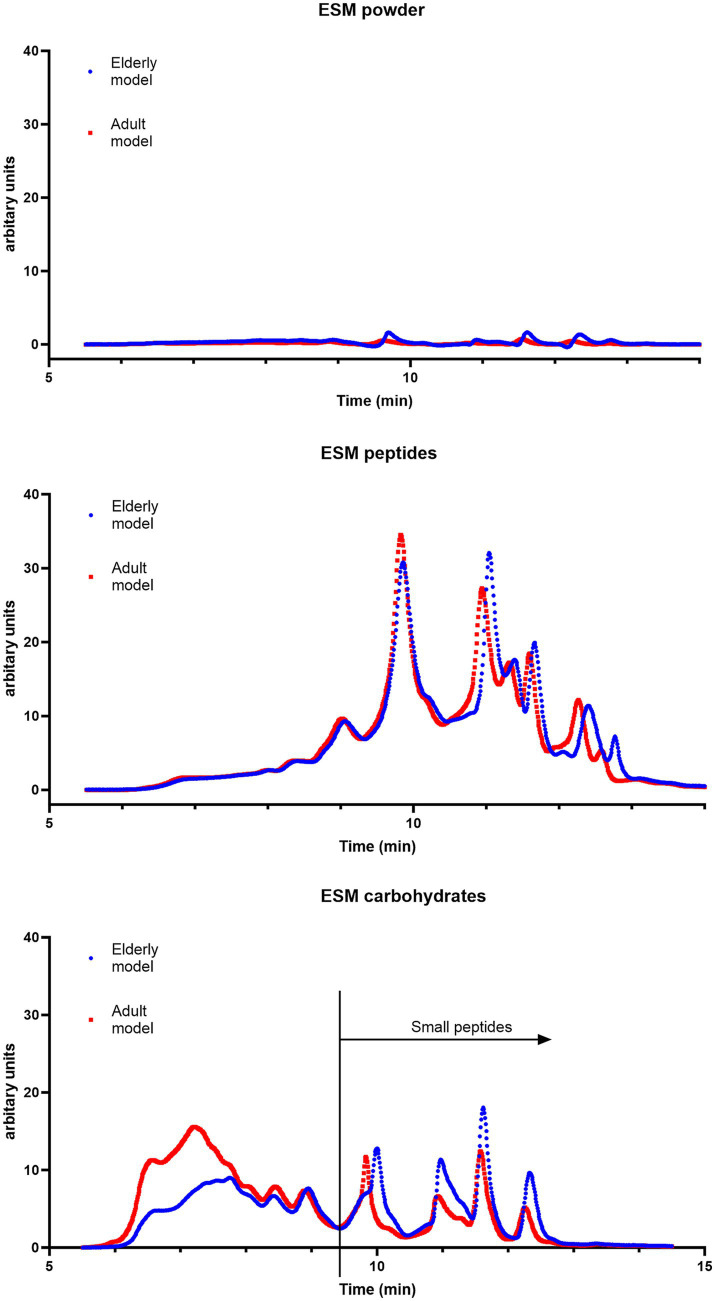
ESM is poorly digested. SEC chromatograms of ESM powder, ESM peptides (alcalase hydrolysate), and ESM carbohydrates (papain hydrolysate fraction) after *in vitro* digestion (80 min in intestinal phase) simulating both adult (blue line) and elderly (red line) gastrointestinal conditions. The X-axis shows the retention time (minutes). The total area under the curve reflects the total amount of soluble proteins/peptides. Small peptides are defined as peptides detected at 9.4 and 15.0 min retention time.

It is known from other studies that protein hydrolysates rich in peptides can stimulate skeletal muscle cells *in vitro* and *in vivo* (55, 56). Complex long-chained carbohydrates such as hyaluronic acid (HA) can revitalize epithelial function (57), indirectly influencing the level of inflammatory cytokines in blood serum. Previous studies demonstrated that micronized ESM (ESM powder) ameliorated intestinal inflammation by facilitating restitution of epithelial injury and increasing muscle mass (25). The *in vitro* protein digestibility of the ESM powder was therefore compared to the digestibility of either a peptide-rich hydrolysate (obtained by alcalase hydrolysis) or a carbohydrate-enriched fraction (GAGs) obtained after papain hydrolysis of the ESM powder (Figure 6). SEC analysis of digested samples clearly showed a higher protein digestibility of the ESM hydrolysate than the powder (Figure 6), mainly due to a significantly better solubility of the hydrolysate in the intestinal phase of the INFOGEST model. Moreover, the ESM hydrolysate was digested equally well in digestive conditions, simulating adult or elderly gastrointestinal states. In contrast to this ESM powder showed decreased digestive hydrolysis under the elderly condition. Like the ESM hydrolysate, the carbohydrate fraction was highly soluble in the intestinal phase. However, the percentage of small peptides was significantly lower, indicating a decline in the rate of hydrolysis, which became even more pronounced under the elderly gastrointestinal condition. Whether the observed decrease was due to indigestible proteins bound to complex carbohydrates needs further investigation.

The Caco2 cell system is a well-characterized intestinal *in vitro* model used to evaluate both the ability of substances to cross the intestinal barrier but also to study possible effects on intestinal inflammation. Previous experiments have demonstrated that adding ESM powder to proliferating Caco2 cells promoted cell viability and lowered the expression of LPS-induced inflammatory cytokines (25). We could not observe any difference in relative gene expression of the tight junction markers Claudin-1 (*CLDN1*) and Tight-junction protein ZO-1 (*TJP1*) in TNFA stimulated Caco2 cells for any of the ESM hydrolysate fractions (Supplementary Figure S5A). For inflammatory cytokines, we could see an increase in *CXCL8* and *IL1B* for the carbohydrate-enriched fraction (Supplementary Figure S5A). No effect could be observed in the gene expression of Heat shock-related 70 kDa protein 2 (*HSPA2*) for any of the fractions. No effect could be observed on the chemokine IL8 secretion, measured by ELISA (Supplementary Figure S5B). The ESM powder could not be transported across the Caco2-cell layer (Supplementary Figure S5C). However, both the peptide and the carbohydrate-enriched fractions were transported across the cell layer. Further evaluation of the skeletal muscle regenerating properties of the different fractions showed that all fractions stimulated bovine skeletal muscle proliferation *in vitro* at higher concentrations (Supplementary Figure S5D). Monitoring the regenerating properties in TNFA stimulated skeletal muscle cells by *PAX7* and *MYOD1* mRNA measurement showed no significant effect of the different fractions, reflecting that the regenerating property was not restored during inflammation (Supplementary Figure S5E). Monitoring stress and inflammation by Nitric oxide synthase (*NOS2*) and *TNFA* at the mRNA level indicated that only the carbohydrate-rich fraction reduced inflammation significantly (Supplementary Figure S5E).

### 3.5 ESM increases mice microbiota diversity and modifies the microbiota composition

**Figure 7:**
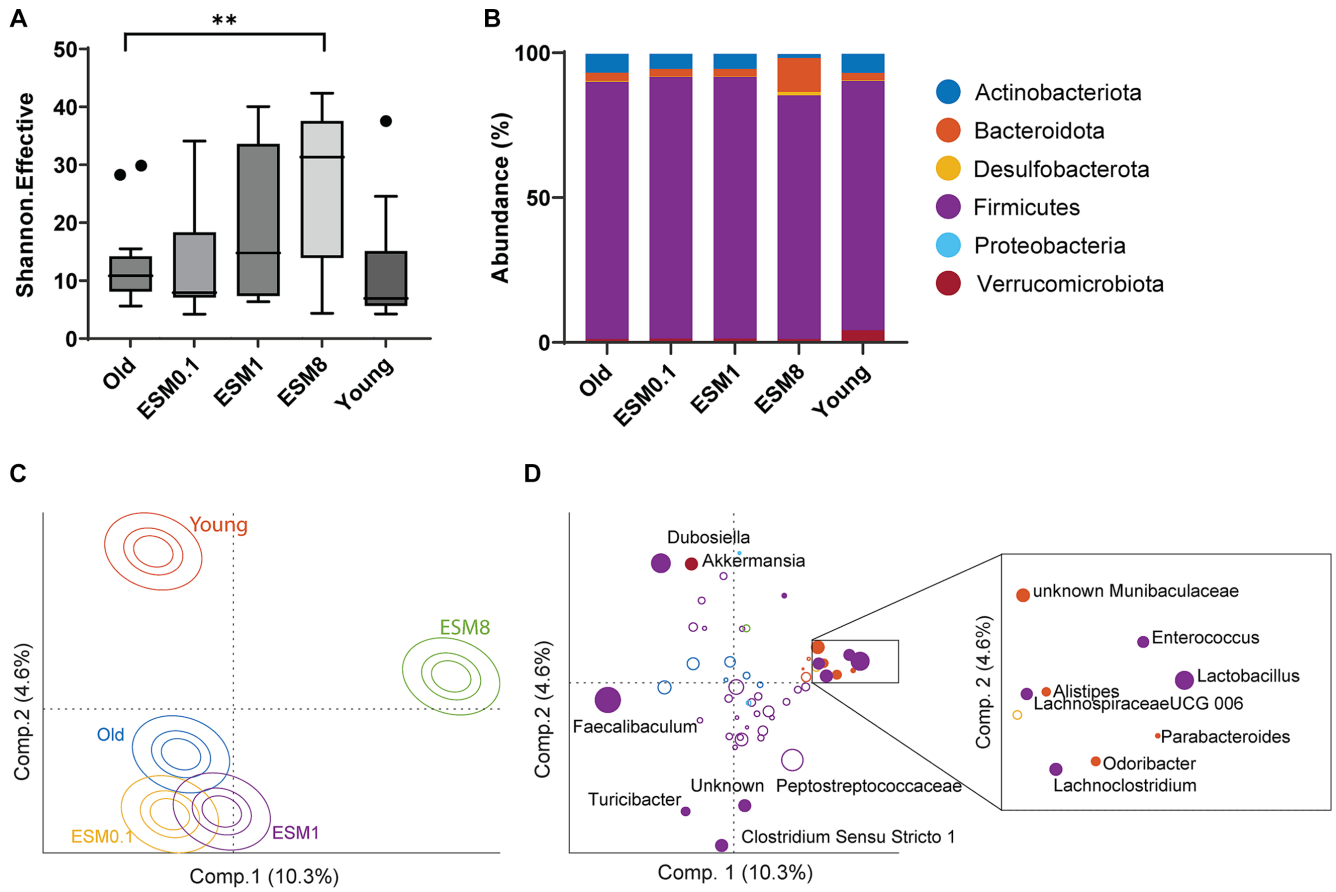
Cecal microbiota effects in mice fed with 8% ESM. **(A)** Microbiota diversity is shown as Shannon effective between the different mouse groups, where asterisk indicates significant difference compared to old control group, **p < 0.01. **(B)** Relative abundance of the bacteria taxa at the phylum level. **(C)** ASCA scores of the compositional differences between mouse groups at the genus level. **(D)** ASCA loadings of bacteria contributing to the variation in microbiota composition, color-coded according to representative phylum described in B. Closed circles indicate bacteria significantly different between mouse groups, with their names indicated. Bacteria significantly enriched with 8% ESM are indicated within the enlarged box.

A healthy gut microbiota closely influences healthy skeletal muscle function (58). Since *in vitro* digestion demonstrated poor digestibility of ESM, particularly under the elderly gastrointestinal condition, the observed effects of ESM on skeletal muscle might be due to undigested ESM reaching the gut and interacting with the gut microbiota. Previous experiments in mice suffering from inflammatory bowel disease and pre-cachexia showed that ESM improved the microbiota dysbiosis, with significant change in *Bacterioidetes, Firmicutes* and Proteobacteria (13, 25). The cecum microbiota was analyzed in all mice groups by 16S rRNA amplicon sequencing, showing that a diet containing 8% ESM had an impact on microbiota diversity and composition (Figure 7). Microbiota diversity (Shannon effective) was significantly increased when old mice were fed with a diet containing 8% ESM compared to the old control group (Figure 7A). At the phylum level, most of the mouse microbiota consist of Firmicutes, and there was a lower abundance of Verrucomicrobiota in all old groups, regardless of an ESM supplemented diet, compared to young mice (Figure 7B). There were also similar levels of Bacteroidota (Bacteroidetes) and Actinobacteriota in young and old mice, however, there was a dose dependent decrease in the proportion of Actinobacteriota in old mice fed with ESM. Interestingly, we also observed a total lack of Desulfobacterota accompanied with an increase in the proportion of Firmicutes in the microbiota of old mice fed with 0.1 and 1% ESM, while in old mice fed with 8% ESM the proportion of both Bacteroidota and Desulfobacterota were substantially increased compared to the old control group, accompanied with a considerable reduction in Firmicutes (Figure 7B). Compositional differences at the genus level are demonstrated in an ASCA score plot (Figure 7C), and genera associated with the two significant directions (Old-ESM8 vs. Old-ESM0.1-ESM1 vs. Young) are shown in the corresponding loading plot (Figure 7D). Especially *Lactobacillus* was highly dominating with 8% ESM (27%) compared to the old control group (5%). Other genera enhanced with 8% ESM were *Enterococcus*, *Muribaculaceae* (previously Bacteroidales S24–7), *Lachnoclostridium, Lachnospiraceae* UCG-006, *Odoribacter, Alistipes*, and *Parabacteroides*. Compared to the old control mice, *Faecalibaculum* was dramatically reduced in 8% of ESM mice (from 47 to 3%). The young mice group contained a relatively higher abundance of *Dubosiella* and *Akkermansia*. Finally, we checked for associations between microbiota (at genus level) and the most important molecular hallmarks of aging and inflammatory markers by PLS regression with full cross validation. The only significant association was found between microbiota and the average area of muscle fibers. However, this association was solely attributed to differences between old and young mice (data not shown).

## 4 Discussion

This study demonstrates that micronized ESM (ESM powder) attenuates skeletal muscle aging in mice. Intake of ESM in elderly mice impacted and attenuated several well-known hallmarks of aging, such as a reduction in the number of muscle fibers, the appearance of centronucleated fibers, a decrease in type IIa/IIx fiber type proportion, reduced gene expression of satellite cell markers *Sdc3* and *Pax7* and increased gene expression of the muscle atrophy marker *Fbxo32.* Similarly, a transition toward the phenotypic characteristics of young mice was also observed for old mice fed with ESM for several proteins associated with energy metabolism (mitochondrial pyruvate dehydrogenase isoforms), protein synthesis (AA-synthesis carrier protein A2A4P3), and myotube development (Nascent polypeptide-associated complex subunit alpha, muscle-specific form, NACAM). The findings in skeletal muscle coincided with an increased gut microbiota diversity and changed the microbiota composition in old mice fed with highest dose of ESM. ESM in the diet also lowered the expression of the inflammation marker TNFA in the aged mice and *in vitro* in THP-1 macrophages. In the human pilot study, the intake of ESM capsules significantly reduced the inflammatory marker CRP. Our data show that the digestion properties of ESM *in vitro* are low.

Skeletal muscle mass is maintained by balancing protein synthesis and degradation. A shift in this balance, with a more significant protein degradation than synthesis, can result in muscle atrophy. Muscle atrophy includes enhanced degradation of proteins via the autophagosome and ubiquitin-proteome system, with F-box only protein 32 (*Fbxo32*) and E3 ubiquitin-protein ligase TRIM63 (*Trim63*) ligases directing the polyubiquitination of proteins to target them for proteolysis by the 26S proteasome and the ubiquitin-mediated protein degradation pathway (59). We did observe a significant change in the mRNA expression pattern of the atrophy genes (*Fbxo32* and *Trim63*) in the TA muscle of mice fed with a diet containing 8% ESM, comparable to the expression pattern observed in young mice. In support of this, the number of myofibers was also higher in the old group fed with 8% ESM. Proteomics data also showed a shift toward increased AA-synthesis (AA-synthesis carrier protein A2A4P3) and potentially increased myotube development (muscle-specific transcription factor NACAM). However, no significant difference in skeletal muscle mass or CSA with or without ESM supplementation was observed, as expected from the increase in AA synthesis. The distribution pattern of minimal Feret’s diameter indicated smaller myofibers in old mice, as expected, and the 8% ESM group (Figure 3B) compared to young mice, reflecting previous cycles of degeneration and regeneration (4). Our mRNA and histology data indicate that biological processes were initiated to restore muscle mass in the 8% ESM group. Another study revealed a reduction of the total number of fibers already in 18–19 months-old mice, albeit with no effect on muscle mass, which aligns with our data (4). They also observed an intermediate compensatory hypertrophy of the remaining fibers (increased CSA minimal Feret’s diameter) followed by an atrophy. A shift toward an increased AA -synthesis and myotube development by our proteomics data could therefore reflect intermediate hypertrophy activity ongoing at 24 months. In younger adults, the response to anabolic stimuli, such as mechanical forces or nutrition, promotes muscle protein synthesis and inhibition of muscle protein degradation. In older age, factors driving anabolic resistance are lost, and inflammation, inactivity, lipid infiltration, and AA sensing have been suggested as some contributing factors (60). It has been proposed that a reduction in AA delivery to the skeletal muscle may impair intracellular signaling and, hence, protein synthesis (61).

SDC3 knock-out mice exhibit aberrant phenotype with centrally nucleated myofibers (62), similar to the phenotype associated with elderly mice in our study. Also, we show that the gene expression of *Sdc3* was higher in young mice compared with the elderly. Interestingly, in our study, the gene expression of *Sdc3* and *Pax7* in old mice fed with 8% ESM showed similar expression levels as young mice, suggesting that ESM impacted activation of MuSCs. We and others have demonstrated that SDC4 plays a prominent role in the early commitment of myogenesis with the fusion of myoblast cells (63). No differences in gene expression level of *Sdc4* or the myogenesis markers, *Myod1*, *Mstn*, and *Myog,* were observed in TA muscle of old mice fed with ESM, reflecting most likely less effect of ESM intake on skeletal muscle regeneration in old mice compared to young mice. In support of this finding, we also showed *in vitro* that TNFA stimulated MuSCs were unable to restore normal function when treated with ESM powder.

Loss of muscle strength and variance in energy metabolism, including both cytosolic and mitochondrial bioenergetics, is often observed in aging. We observed a modest but significant restoration of grip strength during aging when old mice were fed with a diet containing 8% ESM from week 1 to week 9. In a model of murine cachexia in young mice, 8% ESM supplementation improved muscle proteins associated with striated muscle contractions (13). Interestingly, skeletal muscle strength has also been correlated with inflammation markers of skeletal muscle and the circulating level of cytokines. A study of community-dwelling older men showed a significant relationship between gene expression of Myostatin (*MSTN*), circulating cytokines, and grip strength (*IL6*, *TNFA*, and *IL1R1*), serum IL6 and TNFA, and grip strength (64). This is partly in line with our data, where we observed a significant reduction of serum TNFA at end-point measurements in the mice fed with 8% ESM. In contrast, no effect on inflammatory mRNA markers was observed in skeletal muscle. We only investigated the mRNA expression pattern at the end-stage point (10 weeks) and not during the different grip-strength intervals, which could explain this discrepancy. However, gene expression of cytokines in skeletal muscle may not reflect an increase in circulating cytokine concentrations. After exercise, the gene expression of *TNFA* in skeletal muscle increases without a significant increase in the concentration of TNFA in circulation (65). At the molecular level, we observed that ESM affected muscle fiber types, shifting toward an increased number of IIA/IIX fast-twitch myofibers in the 8% ESM group, mimicking the young group. A positive correlation between fast-twitch type IIA and muscle grip strength has been shown, whereas no such correlation was found for type IIX (66). It has also been reported that skeletal muscle shifts to a more aerobic-oxidative, slower-twitching type I fibered population in aging (67). TA skeletal muscle predominantly comprises type II fibers. Among aerobic mitochondrial markers, pyruvate dehydrogenase is reduced during aging (3). Our proteomics data revealed a shift in mitochondrial oxidative metabolism in old mice fed with 1 and 8% ESM, with increased expression of pyruvate dehydrogenase.

Our data demonstrate possible immune-modulating properties of micronized ESM on circulating cytokine levels. In the mouse trial, where old mice mimicked human age 50 to 70 + years, a daily intake of 8% ESM attenuated inflammation. In our small-scale, preliminary human study (using 500 mg/day), no effect on circulating TNFA level was obtained, which was unexpected in accordance with the mice trial. This most likely indicates that intake of more than 500 mg ESM per day would be necessary to impact the TNFA level in humans. Interestingly, we could measure a reduction in another low-grade inflammation marker, C-reactive protein (CRP), in the small-scale, preliminary human study with older adults. A meta-study performed by Neale et al. showed that consumption of a healthy dietary pattern was associated with a significant reduction in CRP. In contrast, the study did not indicate any reductions in other biomarkers, such as TNFA (68). Although high CRP levels are common in elderly with low muscle mass (69), our small-scale, preliminary study did not observe any effect on skeletal muscle function (Supplementary Table 2). A full-scale clinical trial with a higher ESM dose over a longer time would be relevant in the future. Our human small-scale, preliminary study indicate the potential of ESM to affect the level of circulating pro-inflammatory cytokines in the blood. The mechanism of how micronized ESM acts on circulating cytokine levels or the skeletal muscle is unclear. *In vitro* digestion of micronized-ESM using the INFOGEST model revealed that this material is poorly digestible and even less digestible under aging conditions. In the mouse trial, we observed a reduction in body weight of old mice fed with an ESM supplemented diet for 10 weeks, despite no difference in the average food between the ESM groups. This is in line with our INFOGEST data revealing poor digestion properties of ESM.

Other mouse studies have also demonstrated that mice fed with an ESM supplemented diet increased their microbiota diversity, as well as the microbiota composition (70, 71). ESM has been shown to improve gut microbiota dysbiosis caused by DSS-induced inflammation (25). It is interesting to note that *Bacteroides* and *Desulfovibrio*, genera of Bacteroidota and Desulfobacterota that ESM enhanced in our mouse study, are bacteria that are affected by glycosaminoglycans (GAGs), such as glucosamine and chondroitin sulfate (72), which are abundant in ESM. Shang et al. (73) also reported that chondroitin sulfate and its oligosaccharide increased the abundance of genera of Bacteroidota, including *Odoribacter Alistipes* and *Lactobacillus* in mice (73), which are also in agreement with the bacteria that increased with 8% ESM. Whether the effect we observed in mice fed with ESM on the gut microbiota is related to the GAG content or not needs to be further elucidated. Perhaps the effect we observed in the skeletal muscle for mice fed with 8% ESM could reflect a microbiota-gut-muscle axis and, with an indirect effect of ESM on skeletal muscle. 8% ESM reflects a physiologically high dose in a mouse trial, and translating these findings to a nutraceutical product for the human market with an effect on skeletal muscle and microbiota should be done with caution. Also, we cannot exclude that some of the observed effects seen on the skeletal muscle is due to transport of short peptides of digested ESM across the intestinal layer into blood plasma, as our *in vitro* data indicated increased paracellular transport of micronized-ESM and GAG-ESM on paracellular transport without compromising epithelial integrity.

In conclusion, our results suggest that micronized ESM, a natural, low-cost biomaterial, is attractive as a nutraceutical candidate due to its effects on markers of skeletal muscle aging, inflammation and gut microbiota.

## Data availability statement

Sequence data has been uploaded to Sequence Read Archive (SRA) in NCBI and linked to BioProject PRJNA1054009.

## Ethics statement

The studies involving humans were conducted according to the guidelines in the Declaration of Helsinki. All participants gave written informed consent, and the Regional Ethics Committee for Medical Research in Southeast Norway approved the study. The study was registered at ClinicalTrials.gov (ClinicalTrials.gov Identifier: NCTNCT04606628). The studies were conducted in accordance with the local legislation and institutional requirements. The participants provided their written informed consent to participate in this study. The animal study was approved by the European guidelines for the care and use of laboratory animals (European Directive 2010/63/EU) and the Norwegian national guidelines for animal welfare. The protocol was approved by the Norwegian Food Safety Authority (FOTS ID 21576), and the experiment was conducted in the animal facility at the Faculty of Chemistry, Biotechnology and Food Science, Norwegian University of Life Sciences (NMBU; Ås, Norway). The study was conducted in accordance with the local legislation and institutional requirements.

## Author contributions

SBR: Conceptualization, Data curation, Formal analysis, Visualisation, Methodology, Writing-original draft, Writing-review & editing, Funding acquisition. EVK: Conceptualization, Data curation, Formal analysis, Visualisation, Methodology, Writing-original draft, Writing-review & editing, Funding acquisition. IR: Data curation, Formal analysis, Methodology, Writing-review & editing. NTS: Data curation, Formal analysis, Methodology, Writing-review & editing. VH: Data curation, Formal analysis, Methodology, Writing-review & editing. SDCR: Data curation, Formal analysis, Methodology, Writing-review & editing. SB: Data curation, Formal analysis, Methodology, Writing-review & editing. UB: Data curation, Formal analysis, Methodology, Writing-review & editing. AR: Data curation, Formal analysis, Methodology, Writing-review & editing. PB: Data curation, Formal analysis, Methodology, Writing-review & editing. NA: Data curation, Formal analysis, Methodology, Writing-review & editing. IM: Data curation, Formal analysis, Methodology, Writing-review & editing. HA: Data curation, Formal analysis, Methodology, Writing-review & editing. SU: Conceptualization, Methodology, Data curation, Formal analysis, Writing-review & editing, Funding acquisition. KBH: Conceptualization, Methodology, Data curation, Formal analysis, Writing-review & editing, Funding acquisition. HC: Conceptualization, Methodology, Data curation, Formal analysis, Writing-review & editing, Funding acquisition. BK: Conceptualization, Methodology, Data curation, Formal analysis, Writing-review & editing, Funding acquisition. MEP: Conceptualization, Visualisation, Methodology, Writing-original draft, Writing-review & editing, Funding acquisition, Project Administration.

## Footnotes

^1^https://github.com/Lagkouvardos/Rhea
