# Supplementary material for: Dietary intake of micronized avian eggshell membrane in aged mice reduces circulating inflammatory markers, increases microbiota diversity, and attenuates skeletal muscle aging
Source: Front Nutr. 2024 Jan 15;10:1336477. doi: 10.3389/fnut.2023.1336477 (PMC10822908; doi:10.3389/fnut.2023.1336477)
Supplement: Supplementary file 1 [file Data_Sheet_1.docx]

# **Supplementary data for:**

**Dietary intake of micronized avian eggshell membrane in aged mice reduces circulating inflammatory markers, increases microbiota diversity, and attenuates skeletal muscle ageing**

**Sissel B. Rønning^1^, Harald Carlsen^2^, Sérgio D. C. Rocha^3^, Ida Rud^1^, Nina T. Solberg^1^, Vibeke Høst^1^, Eva Veiseth-Kent^1^, Henriette Arnesen^4^, Silje Bergum^1^, Bente Kirkhus^1^, Ulrike Böcker^1^, Nada Abedali^5^, Amanda Rundblad^5^, Pia Bålsrud^5^, Ingrid Måge^1^, Kirsten B. Holven^5,6^, Stine Ulven^5^, Mona E. Pedersen^1*^**

^1^Nofima AS, Food Division, Ås, Norway, Norway.

^2^ Faculty of Chemistry, Biotechnology and Food Science, Norwegian University of Life Sciences, Ås, Norway

^3^ Faculty of Biosciences, Norwegian University of Life Sciences, Ås, Norway

^4^ Faculty of Veterinary Medicine, Norwegian University of Life Sciences, Ås, Norway

^5^ Department of Nutrition, Institute of Basic Medical Sciences, University of Oslo, Norway

^6^ National Advisory Unit on Familial Hypercholesterolemia, Department of Endocrinology, Morbid Obesity and Preventive Medicine, Oslo University Hospital, Norway,


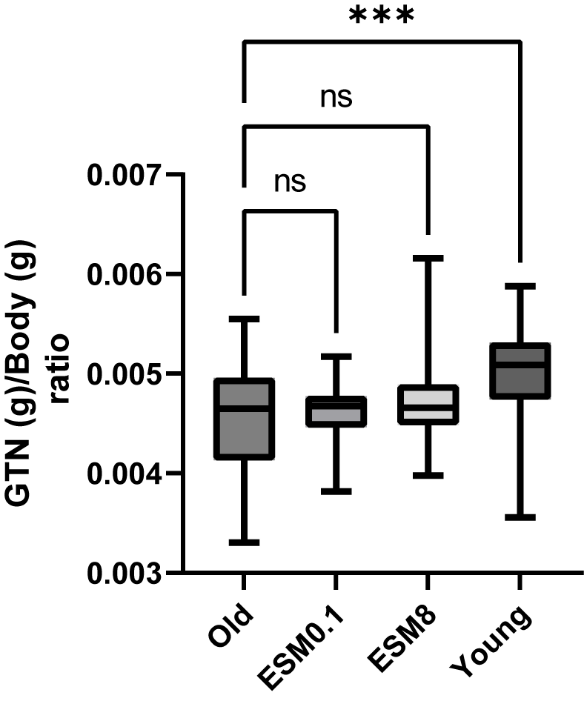


**Figure S1:** Graph shows the ratio between GTN muscle weight (in grams) relative to the total body weight. Asterisks indicate significant differences from old mice, determined by Brown-Forsythe and Welch ANOVA using Dunnett’s multiple comparison test, ***p<0.001.


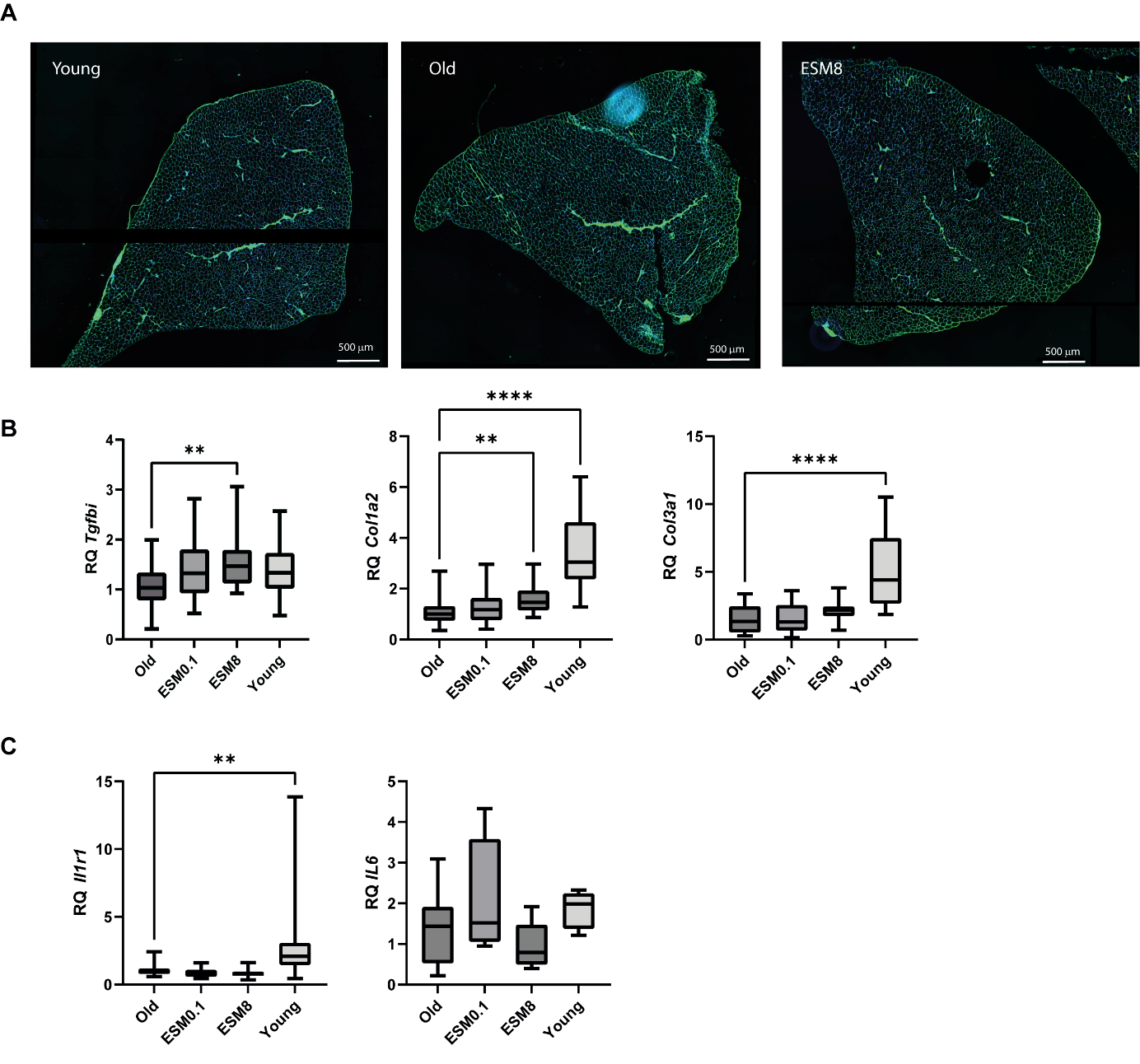


**Figure S2:** Old mice have reduced expression of ECM components and inflammatory markers but have no sign of visible fibrosis. A) The lectins in the connective tissue of TA were stained with Wheat germ agglutinin WGA (green). The cell nuclei were counterstained with Hoechst (blue). Scalebar as indicated. B) Bars show the relative gene expression of the fibrosis marker *Tgfb1*, together with the relative gene expression of *Coll1a2* and *Col3a1* in the TA muscle of old mice fed with or without ESM supplement compared to the average mean RQ value in young mice. C) Bars show the relative gene expression of *IL1r1*, and *IL6* in TA muscle of old mice with or without ESM compared with the average mean RQ value of young mice. Asterisks indicate significant differences from old mice, determined by Brown-Forsythe and Welch ANOVA using Dunnett’s multiple comparison test, **p<0.01, ***p<0.001 ****p<0.0001.


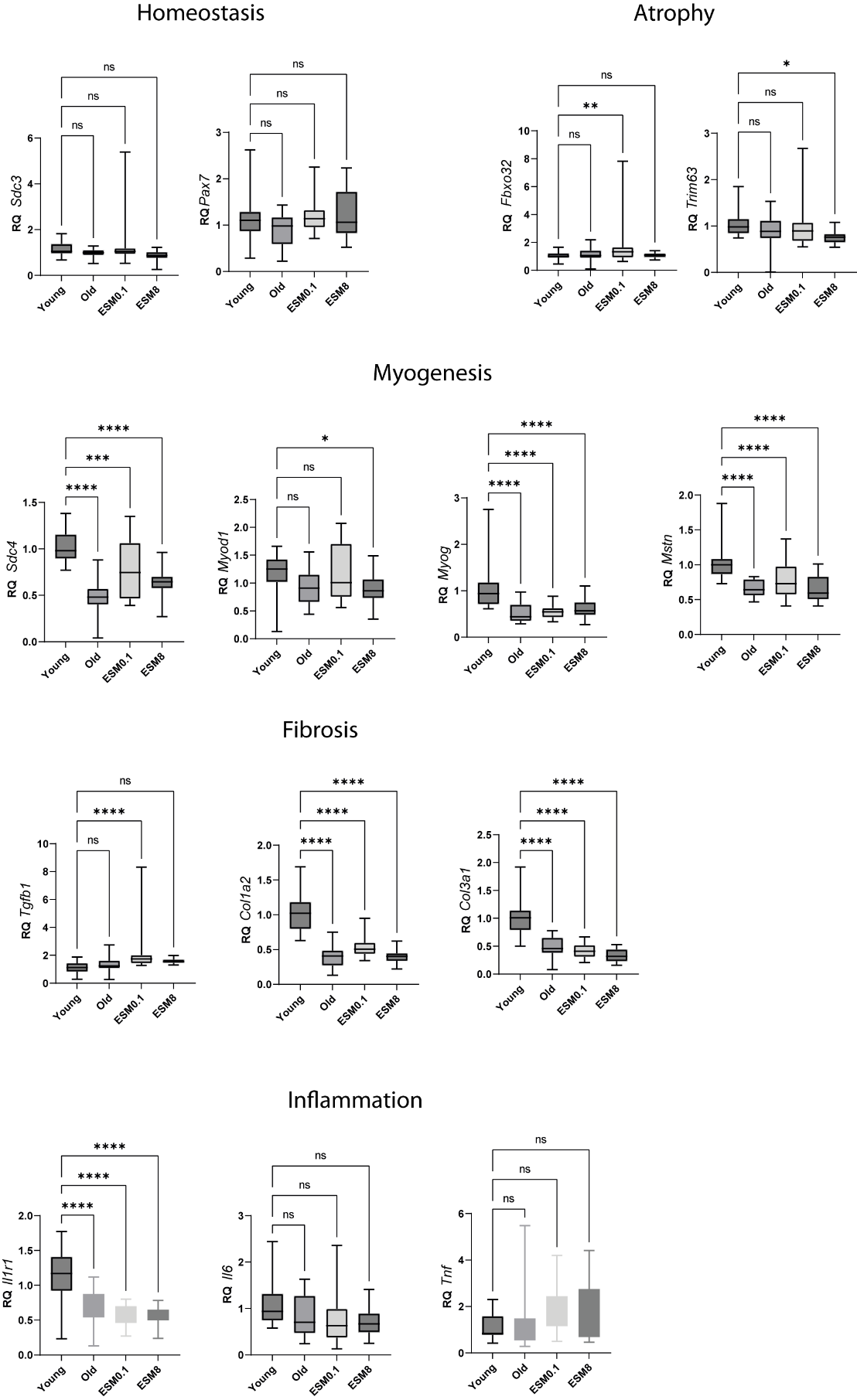


**Figure S3:** Bars show the relative gene expression of homeostasis markers *Sdc3* and *Pax7*, atrophy markers *Fbxo32* and *Trim63*, myogenesis markers *Sdc4*, *Myod1*, *Myog* and *Mstn*, fibrosis marker *Tgfb1*, together with the relative gene expression of *Coll1a2* and *Col3a1* and inflammation markers *Il1r1*, *Tnf* and *Il6* in the GTN muscle of old mice fed with or without ESM supplement compared to average mean RQ value of young mice. Asterisks indicate significant differences compared to young mice, determined by Brown-Forsythe and Welch ANOVA using Dunnett’s multiple comparison test, *p<0.05 **p<0.01, ***p<0.001 ****p<0.0001.


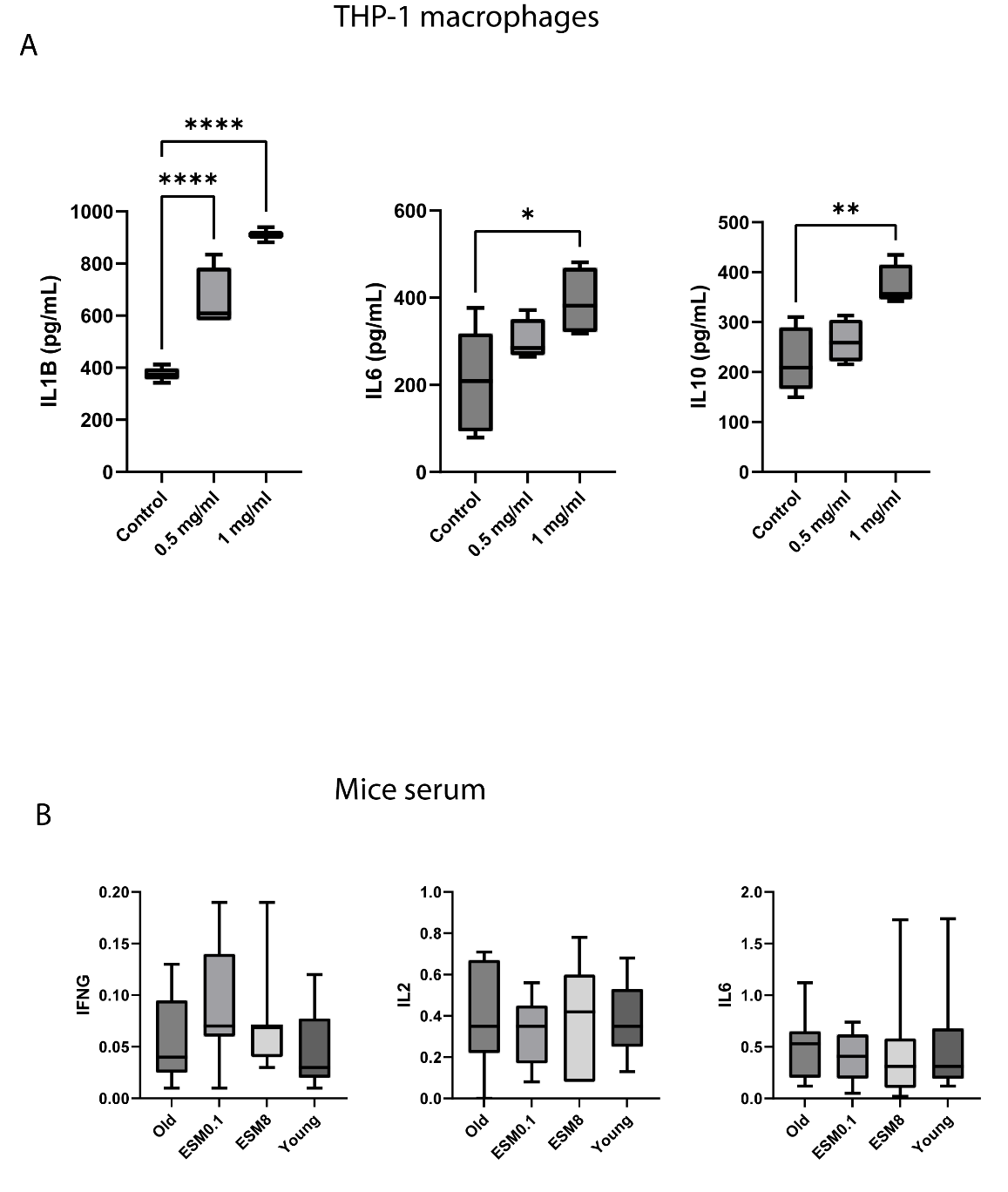


**Figure S4:** A) PMA-differentiated THP-1 cells were incubated with 0.5 ng/ml LPS alone (control) or combined with either 0.5 or 1 mg/ml ESM for 20 h at 37°C. The cell media were collected, and ELISA determined the level of cytokines. The results are presented as mean ± SEM protein level (n=3). (*p<0.05, **p<0.01 or ****p<0.0001 indicate a statistically significant change between control and ESM treated cells, determined by unpaired t-test). B) IFG, IL2 and IL6 levels (pg/mL) in serum samples collected from old mice supplemented with 0, 0.1, 1 and 8% ESM for 10 weeks and young mice (3 months) fed without ESM were measured by Multiplex ELISA. The results are presented as mean ± SEM of all mice within each group (n=15). No significant differences in serum level of these cytokines between the mice groups compared to old mice were detected, determined by Brown-Forsythe and Welch ANOVA using Dunnett’s multiple comparison test, *p<0.05 **p<0.01, ****p<0.0001.


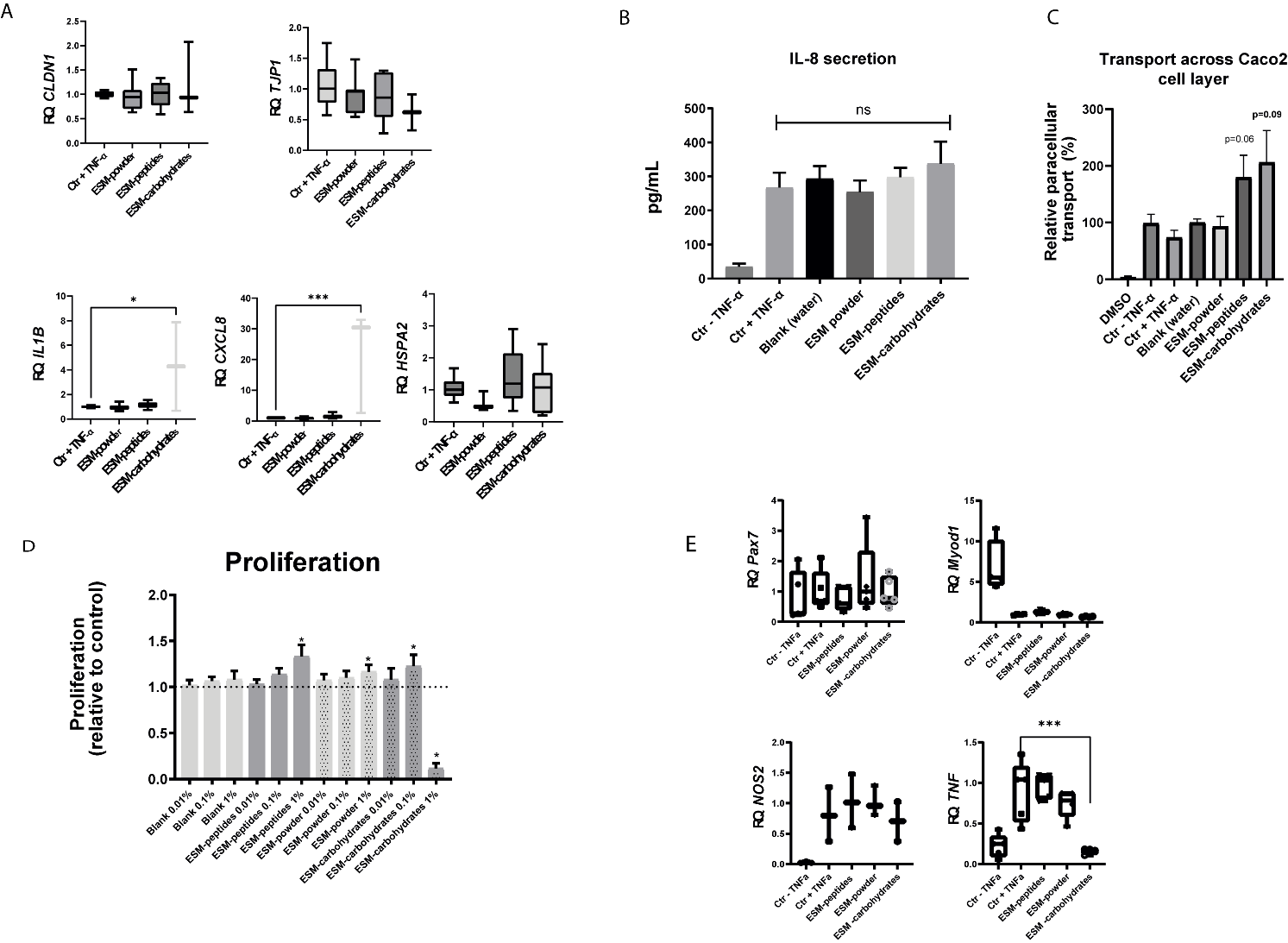


**Figure S5:** A) Bars show gene expression of tight-junction proteins *CLDN1* and *TJP1*, heat shock protein A (*HSPA2*), and cytokines *IL1B* and *CXCL8* in TNFA-treated Caco2 cells. (*p<0.05, **p<0.01 or ****p<0.0001 indicate a statistically significant change between control and ESM treated cells, determined by unpaired t-test). B) Bars demonstrate IL8 secretion in TNFA treated Caco2 cells, with or without digested ESM fractions. C) Paracellular transport across the Caco2 cell layer. D) Dose-response effect on bovine skeletal muscle proliferation after 2 days of ESM supplementation, compared with control cells (treated with digested water control). The data is presented as the average of at least three independent cell culture experiments seeded out in triplicates, +/- SEM. E) Bars show gene expression of the *Pax7*, *Myod1*, *NOS2* and *TNFA* in TNFA-treated inflammatory bovine skeletal muscle cells, with or without ESM-fractions, compared to the average mean of control cells treated with TNFA alone. Asterisks indicate significant differences in treated cells compared with control cells, statistics assessed by one-way ANOVA with Dunnett’s multiple comparison test, *p<0.05 **p<0.01, ***p<0.001 ****p<0.0001.

**Table S1** Baseline characteristics of participants in placebo and ESM groups of the preliminary small-scale human trial

|  | Placebo (n = 19)* | ESM (n = 19)* |
| --- | --- | --- |
| Male, n (%) | 4 (21.1) | 3 (15.8) |
| BMI (kg/m^2^) | 26.2 (3.3) | 25.4 (3.0) |
| Systolic BP (mmHg) | 124.5 (16.7) | 133.6 (18.9) |
| Diastolic BP (mmHg) | 68.0 (10.0) | 70.4 (7.9) |
| Glucose (mmol/L) | 5.46 (0.54) | 5.07 (0.36) |
| TG (mmol/L) | 1.3 (0.3) | 1.0 (0.3) |
| Total-C (mmol/L) | 5.8 (1.0) | 5.9 (1.0) |
| HDL-C (mmol/L) | 1.8 (0.5) | 2.0 (0.5) |
| LDL-C (mmol/L) | 3.7 (1.1) | 3.7 (1.1) |
| microCRP (mg/L) | 1.4 (1.0) | 1.7 (1.0) |
| Non-smoker, n (%) | 19 (100) | 19 (100) |

* Numbers are mean (SD) unless otherwise indicated

**Table S2** Secondary endpoint measurements of skeletal muscle performance in the preliminary small-scale human trial

|  |  | **Placebo** | | |  |  | **Intervention** | | | |
| --- | --- | --- | --- | --- | --- | --- | --- | --- | --- | --- |
|  |  | Baseline |  | End of study |  |  | Baseline |  | End of study | P-value* |
|  | n | mean | n |  |  | n | mean | n | mean |  |
| Systolic BP (mmHg) | 19 | 124 | 19 | 121 |  | 19 | 134 | 19 | 125 | 0,16 |
| Diastolic BP (mmHg) | 19 | 68 | 19 | 66 |  | 19 | 70 | 19 | 68 | 0,83 |
|  |  |  |  |  |  |  |  |  |  |  |
| Weight (kg) | 19 | 72,57 | 19 | 72,54 |  | 19 | 72,2 | 19 | 71,69 | 0,12 |
| Waist circumference (cm) | 19 | 91,2 | 19 | 91,6 |  | 19 | 89,4 | 19 | 87,5 | 0,1 |
| Grip strength (dominant, kg) | 19 | 25,5 | 19 | 25,3 |  | 19 | 24,4 | 19 | 23,1 | 0,23 |
| Grip strength (nondominant, kg) | 19 | 24,3 | 19 | 24,1 |  | 19 | 21,3 | 19 | 21,6 | 0,46 |
| Balance | 19 | 3,9 | 19 | 3,8 |  | 19 | 3,7 | 19 | 3,8 | 0,27 |
| Galt speed | 19 | 4 | 19 | 4 |  | 19 | 4 | 19 | 4 |  |
| Chair test | 19 | 3,7 | 19 | 3,7 |  | 19 | 3,8 | 19 | 3,9 | 0,68 |
| SPPB score | 19 | 11,6 | 19 | 11,3 |  | 19 | 11,5 | 19 | 11,7 | 0,14 |
| Creatine (μmol/L) | 19 | 72,1 | 19 | 71,8 |  | 19 | 68,2 | 19 | 67,2 | 0,68 |
| eGFR (mL/min/1.73m2 | 19 | 76,3 | 19 | 76,4 |  | 19 | 78,2 | 19 | 79,1 | 0,64 |
| Urea (mmol/L) | 19 | 7 | 19 | 6,7 |  | 19 | 6,1 | 19 | 5,9 | 0,95 |
| ASAT (U/L) | 19 | 19,4 | 19 | 18,4 |  | 19 | 23,8 | 19 | 22,2 | 0,7 |
| ALAT (U/L) | 19 | 25,2 | 19 | 26,2 |  | 19 | 24,3 | 19 | 23,7 | 0,33 |
| gammaGT (U/L) | 19 | 24,5 | 19 | 25,9 |  | 19 | 34,2 | 19 | 31,5 | 0,17 |
| Glucose (mmol/L) | 19 | 5,5 | 19 | 5,4 |  | 19 | 5,1 | 19 | 5 | 0,93 |
| Insulin (mmol/L) | 19 | 64,8 | 19 | 69,7 |  | 19 | 47,1 | 19 | 53,1 | 0,9 |
| TG (mmol/L) | 19 | 1,3 | 19 | 1,2 |  | 19 | 1 | 19 | 1,2 | 0,1 |
| Total-C (mmol/L) | 19 | 5,8 | 19 | 5,7 |  | 19 | 5,9 | 19 | 5,8 | 0,78 |
| HDL-C (mmol/L) | 19 | 1,8 | 19 | 1,8 |  | 19 | 2 | 19 | 1,9 | 0,92 |
| LDL-C (mmol/L) | 19 | 3,7 | 19 | 3,7 |  | 19 | 3,7 | 19 | 3,6 | 0,5 |
| apoA1 (g/L) | 19 | 1,6 | 19 | 1,6 |  | 19 | 1,6 | 19 | 1,6 | 0,88 |
| apoB (g/L) | 19 | 1 | 19 | 1 |  | 19 | 1 | 19 | 1 | 0,22 |
|  |  |  |  |  |  |  |  |  |  |  |
| Fat mass (kg) | 19 | 28,71 | 19 | 28,92 |  | 19 | 28,72 | 19 | 28,63 | 0,37 |
|  |  |  |  |  |  |  |  |  |  |  |
| Fat free mass (kg) | 19 | 43,77 | 19 | 43,63 |  | 19 | 43,48 | 19 | 43,06 | 0,4 |
|  |  |  |  |  |  |  |  |  |  |  |
| Skeletal muscle mass (kg) | 19 | 19,79 | 19 | 19,63 |  | 19 | 19,23 | 19 | 19,08 | 0,98 |
| Viceral fat (kg) | 18 | 2,47 | 18 | 2,36 |  | 18 | 2,13 | 18 | 2,01 | 0,96 |
| ***P-value from regression analysis of the difference in change between placebo and intervention from baseline to end of study.** | | | | | | | | | | |
